# LRP1 loss in airway epithelium exacerbates smoke-induced oxidative damage and airway remodeling

**DOI:** 10.1016/j.jlr.2022.100185

**Published:** 2022-02-21

**Authors:** Itsaso Garcia-Arcos, Sangmi S. Park, Michelle Mai, Roger Alvarez-Buve, Lillian Chow, Huchong Cai, Nathalie Baumlin-Schmid, Christina W. Agudelo, Jennifer Martinez, Michael D. Kim, Abdoulaye J. Dabo, Matthias Salathe, Ira J. Goldberg, Robert F. Foronjy

**Affiliations:** 1 Departments of Medicine and Cell Biology, SUNY Downstate Medical Center, New York, NY, USA; 2 Respiratory Department, Hospital University Arnau de Vilanova and Santa Maria, IRB Lleida, University of Lleida, Lleida, Catalonia, Spain; 3 Department of Internal Medicine, University of Kansas Medical Center, Kansas City, KS, USA; 4 Department of Medicine, NYU Langone School of Medicine, New York, NY, USA

**Keywords:** lipoprotein receptors, cell biology, ApoE, receptors, inflammation, animal models, epithelial cells, lung disease, BAL, bronchoalveolar lavage, COPD, chronic obstructive pulmonary disease, CPFE, combined pulmonary fibrosis and emphysema, CSE, cigarette smoke, ECM, extracellular matrix, HBEC, human bronchial epithelial cell, IC, inspiratory capacity, IPA, ingenuity pathway analysis, KC, keratinocyte chemoattractant, LRP1, LDL receptor-related protein 1, MMP, matrix metalloprotease, ns, not significant, PBST, PBS containing Tween-20, qPCR, quantitative PCR, ROS, reactive oxygen species, TGF-β, transforming growth factor beta

## Abstract

The LDL receptor-related protein 1 (LRP1) partakes in metabolic and signaling events regulated in a tissue-specific manner. The function of LRP1 in airways has not been studied. We aimed to study the function of LRP1 in smoke-induced disease. We found that bronchial epithelium of patients with chronic obstructive pulmonary disease and airway epithelium of mice exposed to smoke had increased LRP1 expression. We then knocked out LRP1 in human bronchial epithelial cells in vitro and in airway epithelial club cells in mice. In vitro, LRP1 knockdown decreased cell migration and increased transforming growth factor β activation. Tamoxifen-inducible airway-specific LRP1 knockout mice (club *Lrp1*^−/−^) induced after complete lung development had increased inflammation in the bronchoalveolar space and lung parenchyma at baseline. After 6 months of smoke exposure, club *Lrp1*^−/−^ mice showed a combined restrictive and obstructive phenotype, with lower compliance, inspiratory capacity, and forced expiratory volume_0.05_/forced vital capacity than WT smoke-exposed mice. This was associated with increased values of Ashcroft fibrotic index. Proteomic analysis of room air exposed-club *Lrp1*^−/−^ mice showed significantly decreased levels of proteins involved in cytoskeleton signaling and xenobiotic detoxification as well as decreased levels of glutathione. The proteome fingerprint created by smoke eclipsed many of the original differences, but club *Lrp1*^−/−^ mice continued to have decreased lung glutathione levels and increased protein oxidative damage and airway cell proliferation. Therefore, LRP1 deficiency leads to greater lung inflammation and damage and exacerbates smoke-induced lung disease.

The LDL receptor-related protein 1 (LRP1) is a member of the LDL receptor family and performs endocytic and signaling functions. LRP1 was originally identified as a receptor for chylomicron remnants and alpha-2 macroglobulin (1, 2), but many other ligands have been identified and their specific roles and regulation depend on cell type and tissue. Amongst others, extracellular LRP1 ligands include lipoproteins, matrix metalloproteases (MMPs), antiproteases such as tissue plasminogen activator, and extracellular matrix (ECM) components such as fibronectin. Intracellular LRP1 ligands include scaffolding and signaling proteins and membrane coreceptors (3). Some of the functions of LRP1 reflect its role as a lipoprotein receptor, whereas others are more in line with its anti-inflammatory actions mediated by interaction with serpins and tissue inhibitor of metalloproteinases (4, 5).

In the human heart, LRP1 expression was greater with ischemic heart disease, and its activation with a synthetic ligand was cardioprotective in mice subjected to experimental acute myocardial infarction by ischemia-reperfusion (4). Genome-wide association study has linked SNPs in *LRP1* with several diseases, such as abdominal aortic aneurysm, hyperlipidemia, and chronic obstructive pulmonary disease (COPD) (6, 7, 8, 9). Some of these SNPs decrease the mRNA stability and protein expression of LRP1. In smokers and patients with COPD, SNPs in *LRP1* correlate with decreased lung function (9), but the role(s) of LRP1 in normal and pathological pulmonary physiology is relatively unexplored.

Complete deletion of LRP1 in mice results in neonatal death (10), hence tissue-specific knockout mice have been used to decipher the functions of LRP1. Macrophage-LRP1 deficiency increased expression of proinflammatory mediators, whereas also decreasing VLDL uptake and lipid accumulation in vitro (11). In vivo, macrophage LRP1 deficiency worsened atherosclerotic lesions (12, 13). These data suggest that anti-inflammatory roles of LRP1 in macrophages are predominant over its lipid metabolic effects. Liver-specific *Lrp1* knockout mice developed nonalcoholic fatty liver disease (14), an effect opposite to that expected for a receptor known to mediate uptake of chylomicron remnants. Adipose-specific deletion of *Lrp1* delayed postprandial triglyceride clearance and decreased adipogenesis (15), suggesting that it primarily functions as a lipoprotein receptor in adipocytes.

Although LRP1 is highly expressed in lungs and its actions affect inflammation and repair (reviewed in Ref. (16)), the specific cell processes responsible for these effects remain to be defined. One possibility is that LRP1 is required for alveolar macrophages to eliminate pathogens and cell debris (17). Another is that fibroblast LRP1 clears MMP-2 and MMP-9 and protects the integrity of the ECM (18). Another likely site of LRP1 actions in the lung is via its expression in epithelial cells, but the role of LRP1 specifically in airway epithelium has not been reported.

We aimed at elucidating the role of LRP1 in airway epithelium. We found that LRP1 expression was increased primarily in bronchial epithelium of COPD patients and generated club cell-specific tamoxifen-inducible LRP1 knockout mice (club *Lrp1*^−/−^). Club *Lrp1*^−/−^ mice had increased pulmonary inflammation, and in response to chronic cigarette smoke, they exhibited worse pulmonary compliance and greater fibrotic scoring than WT mice. Proteomic analysis of isolated club cells linked this phenotype with cytoskeleton signaling and oxidative damage. We found that club LRP1 expression increases resistance to smoke and likely explains why SNPs in this gene associate with greater COPD in humans.

## Materials and methods

### Generation of club *Lrp1*^−/−^ mice

All experiments involving animals were approved by the IACUC of SUNY Downstate Health Sciences University. Commercially available LRP1^flox/flox^ mice were crossed with Scgbla1-Cre/ER™ mice (The Jackson Laboratory) to generate Scgbla1-Cre/ER^TM^LRP1^flox/WT^ (hemizygous). These mice were crossed again with LRP1^flox/flox^ to generate tamoxifen-inducible Scgbla1-Cre/ER^TM^LRP1^flox/flox^ mice (club *Lrp1*^−/−^). Genotyping was conducted following the protocol from The Jackson Laboratory. Club *Lrp1*^−/−^ mice and their littermate controls LRP1^flox/flox^ (WT) were born at the expected Mendelian ratio and did not show any visible phenotype during postnatal development. Activation of Cre recombinase and subsequent LRP1 loss specifically in club cells was induced by tamoxifen injection for five consecutive days after complete lung maturation, when the mice were 5–7 weeks of age. Different sets of mice were used for pulmonary function testing, tissue collection, and primary cell isolation for proteomic analysis.

### Bronchoalveolar lavage and tissue collection

Mice were anesthetized, the trachea was cannulated, and the heart was PBS-perfused. Bronchoalveolar lavage (BAL) fluid was collected by tracheal instillation of 1 ml PBS through a 20G catheter. Recovered cells were pelleted and counted in a Neubauer counting chamber. An aliquot of the collected cells was stained with QuickDiff, and cell types were counted under a light microscope. Tissues were flash-frozen and stored at −80 C until further analysis. In some experiments, lungs were pressure perfused at 25 cm H_2_O with formaldehyde (19) and used for sectioning and histology.

### Under agarose chemotaxis assay

Immune cells were isolated from mouse femur bone marrow using the protocol by Liu and Quan (20). Briefly, donor mice were anesthetized with isoflurane and euthanized. Both femurs were cut off from the hind legs and muscles, and residual tissues surrounding the femurs were removed. The bone marrow was flushed out from the femurs using DMEM-F12 culture media containing 5% FBS and 1% penicillin/streptomycin. Flushed cells were filtered through 100 μm and 40 μm strainers and centrifuged at 1,500 rpm for 7 min at 4°C. The cell pellets were resuspended in red blood cell lysis buffer for 10 min at room temperature to remove red blood cells. Cells were pelleted at 1,500 rpm for 7 min at 4°C and then counted.

Lungs from WT and club-*Lrp1*^−/−^ mice were collected after BAL and PBS-perfusion through the heart and cut into 1 mm pieces. The chemotaxis assay was performed (21). Briefly, a 6-well plate (35 mm diameter) filled with 2% solidified agarose was prepared by punching each well with three equidistant diameter holes of 3 mm. The isolated bone marrow-derived immune cells (10^6^ cells) were loaded in the center hole, and lung pieces from WT or club-*Lrp1*^−/−^ mice were added to each side hole with culture media. The plate was incubated in a chamber at 37°C, 5% CO_2_ for 24 h, and then fixed with 4% paraformaldehyde in PBS, rinsed with PBS, and air dried. The agarose gel and the cells embedded were stained with Kwik Diff and washed with distilled water. The distances from the center of the hole and the rocket formed by the immune cells migrating toward WT and club-*Lrp1*^−/−^ lungs were measured. The results are reported as mean ± SD.

### Primary cell isolation

Primary airway epithelial cells were isolated from WT and club *Lrp1*^−/−^ mice as in the study by Oreffo *et al.* (22). Briefly, mice were anesthetized with isofluorane, and their trachea was cannulated. The abdominal cavity was opened, and the heart was perfused with PBS until the lungs and liver were free of blood. The lungs were then lavaged through the tracheal cannula with 1 ml saline, followed by protease solution (0.25% crystalline trypsin in 133 mM NaCl, 5.2 mM KCl, 1.89 mM CaCl_2_, 1.29 mM MgSO_4_, 2.59 mM phosphate buffer, pH 7.4, 10.3 mM Hepes buffer, pH 7.4, and glucose 1 mg/ml) and then infused with protease solution according to the standard lung perfusion guidelines by the American Thoracic Society (19). Lungs were maintained filled with protease solution at 37°C for 15 min. Then, lungs and airways were extracted from the chest cavity and dissected in protease solution supplemented with fetal bovine serum. Trachea and major bronchi were removed, and the parenchyma was diced in small pieces, placed in solution B (5.2 mM KCl, 2.59 mM phosphate buffer, pH 7.4, 10.3 mM Hepes buffer, pH 7.4, glucose 1 mg/ml), and manually shaken to release the digested cells. The cell suspension was then filtered through gauze and 100 and 40 μm mesh. The primary cell digest was centrifuged twice at 32 *g* for 6 min at 10°C in 4 ml solution B with 250 μg/ml DNase, and the final pellet was collected.

### Proteomic analysis

Airway epithelial cells collected as aforementioned (biological replicates: N = 3–4 mice/condition) were submitted to Bioproximity LLC (Chantilly, VA), where they underwent standardized proteomic analysis workflow (technical replicates: N = 1 run/sample) as follows. For protein denaturation and digestion, samples were prepared using the filter-assisted sample preparation method (23). Briefly, the samples were suspended in 2% SDS, 50 mM Tris-HCl, pH 7.6, 3 mM DTT, sonicated briefly, and incubated in a ThermoMixer at 40°C, 1,000 rpm for 20 min. Samples were centrifuged to clarify, and the supernatant was transferred to a 30 K Amicon Molecular Weight Cutoff device (Millipore) and centrifuged at 13,000 *g* for 30 min. The remaining sample was buffer exchanged with 8 M urea, 100 mM Tris-HCl, pH 7.6, and then alkylated with 15 mM iodoacetamide. The urea concentration was reduced to 2 M. Samples were digested using trypsin at an enzyme to substrate ratio of 1:40, overnight, at 37°C on the ThermoMixer at 1,000 rpm. Digested peptides were collected by centrifugation. A portion of the digested peptides, about 20 μg, were desalted using C18 stop-and-go extraction tips (24). Briefly, for each sample, a C18 stop-and-go extraction tip was activated with methanol, conditioned with 60% acetonitrile, 0.5% acetic acid followed by 2% acetonitrile and 0.5% acetic acid. Samples were loaded onto the tips and desalted with 0.5% acetic acid. Peptides were eluted with 60% acetonitrile, 0.5% acetic acid, and lyophilized in a SpeedVac (Thermo Savant) to near dryness, approximately 30 min. Each digestion mixture was analyzed by UHPLC-MS/MS. LC was performed on an Easy-nLC 1000 UHPLC system (Thermo). Mobile phase A was 0.1% formic acid in LC-MS grade water (Sigma). Mobile phase B was 99.9% acetonitrile and 0.1% formic acid. The 60 min LC gradient ran from 0% B to 35% B over 45 min and then to 80% B for the remaining 15 min. Samples were loaded directly to the column. The column was 50 cm × 75 um I.D. and packed with 2 micron C18 media (Thermo Easy Spray PepMap). The LC was interfaced to a quadrupole-Orbitrap mass spectrometer (Q-Exactive; Thermo Fisher) via nano-electrospray ionization using a source with an integrated column heater (Thermo Easy Spray source). The column was heated to 50°C. An electrospray voltage of 2.2 kV was applied. The mass spectrometer was programmed to acquire, by data-dependent acquisition, MS/MS from the top 20 ions in the full scan from *m/z* 400 to 1,200. Dynamic exclusion was set to 15 s, singly charged ions were excluded, isolation width was set to 1.0 Da, and full MS resolution was set to 70,000 and MS/MS resolution to 17,500. Normalized collision energy was set to 25, automatic gain control to 2e5, maximum fill MS to 20 ms, maximum fill MS/MS to 60 ms, and the underfill ratio to 0.1%. Mass spectrometer RAW data files were converted to MGF format using msconvert (25). Detailed search parameters are printed in the search output XML files. Briefly, all searches required 5 ppm precursor mass tolerance, 0.01 Da fragment mass tolerance, strict tryptic cleavage, up to two missed cleavages, fixed modification of cysteine alkylation, variable modification of methionine oxidation, and expectation value scores of 0.01 or lower. MGF files were searched using most recent monthly update of the UniProt human sequence library. MGF files were searched using X!!Tandem (26) using both the native (27) and k-score (28) scoring algorithms and by OMSSA (29). MGF files were searched using X!Hunter (30) against the GPM (31) spectral library. The release versions of the peaklist-generating softwares and search engines used were as follows: X! Tandem Vengeance (2015.12.15.2), OMSSA 2.1.9, Open MS 1.11.1, and ProteoWizard 3.0.9283. The sequence library was retrieved on December 19, 2016, and the exact number of entries is unknown. All searches were performed on Amazon Web Services-based cluster compute instances using the Proteome Cluster interface. XML output files were parsed, and nonredundant protein sets were determined using Proteome Cluster (32). MS1-based peak areas were calculated using XCMS (33). Proteins were required to have one or more unique peptides across the analyzed samples with E-value scores of 0.01 or less. Tables with all protein identifications, accession number for UniProt, number of distinct peptides assigned to each protein, coverage of each protein, and spectral counts for each sample are provided in supplemental File S1. Proteins that were identified by a single peptide were removed for the downstream analysis, but the full information is available in MassIVE, with accession number MSV000083163 (ftp://massive.ucsd.edu/MSV000083163).

For comparison between samples, WT mice exposed to room air were used as control group. Only proteins detected in all mice were considered for analysis. Using Proteome Cluster interface, for each protein in the experimental groups, the detection level was normalized to its level in the control group. This value was then expressed as fold change for each condition (WT room air, club *Lrp1*^−/−^ room air, WT smoke, and club *Lrp1*^−/−^ smoke). Proteome data were then analyzed through the use of Ingenuity Pathway Analysis (IPA) software (Qiagen), using the December 2020 release (34). The dataset containing all protein identifiers, relative detection levels, and initially calculated *P* values versus room air control mice was uploaded to the application. The proteins from the dataset were matched with the canonical pathways in the reference set of IPAs. The reference set considered only experimentally observed relationships (highest confidence filter). The significance of the association between the protein dataset and the canonical pathway was measured in two ways: *1*) a ratio of the number of molecules from the dataset that map to the pathway divided by the total number of molecules that map to the canonical pathway is displayed in the figures; *2*) Benjamini-Hochberg correction for multiple comparison tests was applied. LRP1 network (Fig. 4A) was obtained by direct interrogation to the database of IPAs, and the overlapping pathways (Fig. 4C) were limited to the most significant top 10. While significant and nonsignificant changed proteins are reported in the results section, only proteins with statistically significant changes (*P* < 0.05) were used for the pathway analysis discussed.

### Smoke exposure and pulmonary function assessment

Mice were exposed to room air or to daily smoke from 20 cigarettes (4 h per day, 5 days per week) at a total particulate matter concentration of 100 μg/m^3^ in a whole-body exposure chamber (Teague Enterprises) for 6 months. Baseline pulmonary function measurements and airway responses to methacholine were determined with a Scireq Flexivent system (35).

### Histology

The lungs underwent pressure fixation, and fixed tissue was processed for trichrome staining and Ki67 immunohistochemistry by HistoWiz, Inc (NY). Slides were visualized using a brightfield microscope equipped with a 20× objective. Fibrosis was determined by a blind investigator using the Ashcroft index (36) to score 30 different areas in each sample. Scores were averaged, and the means were compared between conditions.

### RNA extraction and quantitative PCR

RNA was extracted from samples using commercially available reagents (Qiagen), and quantitative PCR (qPCR) was performed using SYBR Green fluorophore (Bio-Rad). Primers were designed using OligoPerfect Designer Tool (Invitrogen). Ct data were normalized to actin and analyzed by the ΔΔCt method.

### Western blot

Tissues were homogenized in four volumes of RIPA buffer, and cells were pelleted and disrupted in 300 μl RIPA buffer. Protein concentration in the lysates was determined by the BCA method. Equal amounts of protein were Western blotted into PVDF membranes, blocked with 5% BSA in Tris-buffered saline with Tween-20 overnight at 4°C, and probed with specific antibodies. Proteins were detected by ECL-chemiluminiscence.

### Oxidative stress assessment

Reactive oxygen species (ROS), protein-carbonyl adducts, and glutathione amounts were determined in lung tissue using commercially available kits (Cell Biolabs) and following the manufacturer's instructions. Values were normalized to lung protein concentration or tissue weight as indicated in the figures.

### Cell line culture and transfections

Primary human small airway epithelial cells were purchased from Lonza, cultured as recommended. Human bronchial epithelial cell (HBEC) line was purchased from ATCC, grown in submerged conditions according to the provider's instructions, and used at passage 7 for all experiments. Transfections were performed with siRNA delivered with Hyperfect Plus (Qiagen) for 4 h in Opti-MEM media. Reporter assays for measuring transforming growth factor beta (TGF-β) (through activity of transcription factors mothers against decapentapeglic homologs 2,3 and 4 [SMAD2/SMAD3/SMAD4]) were used following the manufacturer's instructions (Qiagen). After the transfection time, media were replaced for complete culture media including 5% cigarette smoke (CSE) or PBS. Cells were collected or used for experiments 48 h later.

### Analysis of cellular morphometry

Cell cultures were imaged at 40×, 48 h after transfection and treatment with CSE or PBS. Cell observations were performed at identical time points and after plating the same cellular density for both LRP1 and scrambled siRNA-transfected cells. Each condition was tested in triplicate wells, and a minimum of four representative images were collected per well. The number of cells pictured and analyzed was N = 69–235/condition. ImageJ software (Nationl Institutes of Health) was used for the image analysis. First, manual segmentation of the image was performed by delineating the perimeter of each cell with the freehand tool and filling the enclosed area with color. This image was then transformed to binary and converted to a mask. Fractal Analysis plugin (FracLac) was used to calculate the morphometric parameters of the mask scanning of the image with the particle analyzer setting, thus measuring each cell separately. FracLac automatically calculates the smallest convex polygon enclosing each cell (convex hull) and its metrics, including radius, area, perimeter, and others (http://rsb.info.nih.gov/ij/plugins/fraclac/FLHelp/Introduction.htm). The following parameters were calculated: perimeter, maximum span (maximum distance between two points across the cell, as an estimate of the cellular elongation and numerical descriptor of the filopodia), mean radius (of the minimum-sized circular frame enclosing each cell), area (of the enclosing frame), and cellular body density (area of the enclosing frame actually occupied by the cell).

### Cell adhesion to ECM

Experiments were performed (37). Briefly, 96-well plates were coated with 8 μg/cm^2^ collagen overnight at 4°C. Then, plates were washed with PBS and incubated with 2% BSA for 2 h at room temperature. Transfected HBECs grown with 5% CSE or PBS were resuspended in fresh media, seeded, and allowed to adhere for 1 h in the incubator at 37°C and 5% CO_2_. Plates were then washed with PBS three times to remove nonadherent cells and incubated with formaldehyde 4% for 15 min. Fixed cells were stained with 0.2% crystal violet for 45 min, and stain was extracted with 1% SDS. Absorbance was measured at 595 nm.

### Cell migration through ECM

Experiments were performed (37). Briefly, transwell chambers with 8 μm pores were coated with collagen at a density of 8 μg/cm^2^ by treating the bottom membrane of each insert. Transfected HBECs grown with 5% CSE or PBS and resuspended in fresh media were seeded on the top side of the chamber and allowed to migrate toward the bottom chamber, which contained media supplemented with 10% FBS, for 4 h in the incubator at 37°C and 5% CO_2_. Then, plates were placed in cold, and the top membrane of each insert was scrapped to remove nonmigrating cells. Membranes were stained for QuickDiff and inspected under the microscope with a 20× objective. Positively stained cells were counted in three noncontiguous fields for each insert.

### Human bronchial sections and quantification of immunostaining

Human bronchial tissues and HBECs were obtained from organ donors whose lungs were rejected for transplant. Fresh tissue was fixed in 10% formalin, embedded in paraffin, and cut at 5 μm thickness. Sections were deparaffinized in xylene for 10 min, 100% ethanol for 6 min, 95% ethanol for 2 min, 80% ethanol for 2 min, and rinsed in distilled water. Antigen retrieval was performed using trisodium citrate at pH 6 for 30 min in a 98°C water bath. After allowing the sections to cool down to room temperature, they were rinsed in PBS containing 0.05% Tween-20 (PBST) and blocked in 3% BSA in PBS for 1 h at room temperature. LRP1 antibody was added following the manufacturer’s recommendation in 3% BSA and incubated overnight at 4°C on a rocking shaker. Sections were then washed in PBST three times for 10 min and incubated with secondary antibody in 3% BSA for 1 h at room temperature. Sections were washed two times for 10 min in PBST, and Hoechst dye was added in PBST for 10 min. After a final wash in PBST for 10 min, mounting media were added, and the sections were allowed to dry for 24 h before visualization. Images were acquired using a Nikon C2+ confocal microscope equipped with a 20× objective and maintaining identical adjustments for all sections.

### Statistics

Experimental data were analyzed using GraphPad Prism Software (GraphPad Software, Inc). Means were compared by *t*-test when two groups were analyzed and by two-way ANOVA followed by Tukey post-test when four groups were analyzed. For the proteomic dataset, Benjamini-Hochberg correction for multiple comparison tests was applied to determine the pathways involved. The cut-off significance for a level change to be further considered was *P* < 0.05.

### Study approval

Lungs were provided by the Life Alliance Organ Recovery Agency of the University of Miami in Florida. The Institutional Review Board determined that the consent of organ donation for research done by the organ procurement agencies covers research use of this material. Since the material was obtained from deceased individuals with minor and deidentified information, its use does not constitute human subjects research as defined by Code of Federal Regulations 46.102. All animal studies were performed with approval by the IACUC of the State University of New York (SUNY Downstate).

## Results

### COPD and smoke exposure induced LRP1 expression in human airway epithelium

**Fig. 1:**
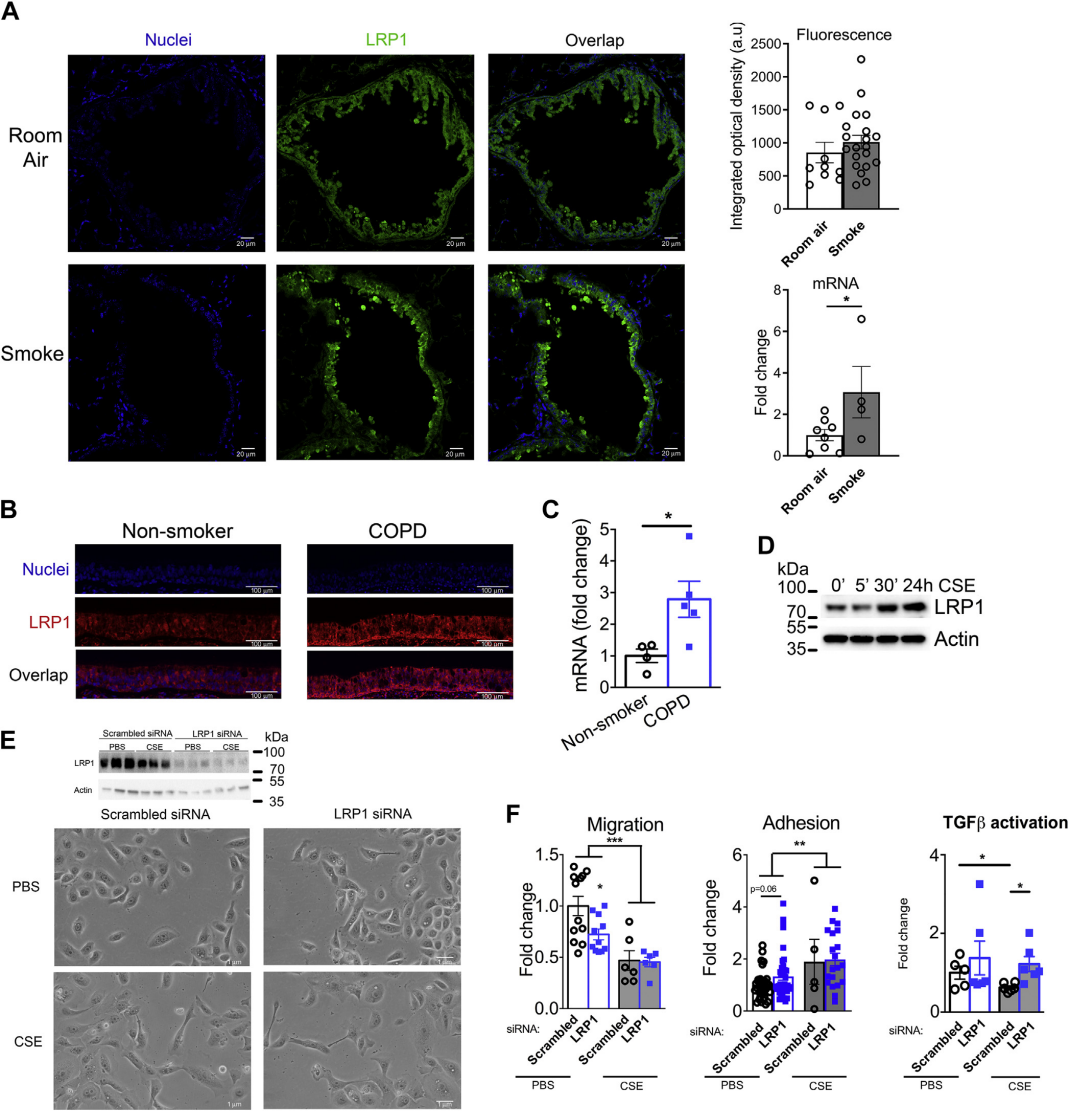
LRP1 expression in human airway epithelium. A: Immunohistochemistry for LRP1 (green) and nuclei (blue) detection in airway epithelium of mice exposed to 6 months of room air or smoke. LRP1 fluorescence optical density and mRNA expression in club cells are shown in the right graphs. B: Representative images of immunohistochemistry for LRP1 (red) and nuclei (blue) detection in human bronchial sections from nonsmokers and COPD donors. C: mRNA expression of *LRP1* in primary human airway epithelial cells isolated from nonsmokers and patients with COPD. D: LRP1 protein expression in human small airway epithelial cells exposed to CSE in culture. E: Light microscopy of HBEC transfected with scrambled or *LRP1*-specific siRNA and cultured with PBS or CSE-supplemented media for 48 h. F: Cell adhesion to collagen-coated wells and migration through a collagen matrix of HBEC and reporter assays for activity of TGF-β promoter in HEBC transfected with scrambled or *LRP1*-specific siRNA and cultured with PBS or CSE-supplemented media for 48 h. N = 4–24/group.  WT;  club *Lrp1*^−/−^ ∗*P* < 0.05, ∗∗*P* < 0.01, and ∗∗∗*P* < 0.001.

LRP1 is widely expressed in the different cell types in human lungs (https://www.proteinatlas.org/ENSG00000123384-LRP1/tissue/lung). In mice, LRP1 showed positive staining in cells in the airway epithelium and parenchyma (Fig. 1A). qPCR of isolated club cells, which in mice, line the bronchi and distal airways together with ciliated cells, showed higher LRP1 expression in mice exposed to 6 months of second-hand smoke than in mice exposed to room air (*P* < 0.05; Fig. 1A). In immunofluorescent studies of human bronchial sections, LRP1 was highly expressed, and its expression was increased in airway epithelial cells from patients with COPD (Fig. 1B). Consistently with a role for LRP1 in response to airway disease, primary HBECs isolated by bronchial brushing had ∼3-fold increase in LRP1 mRNA in patients with COPD (*P* < 0.05) (Fig. 1C). As a model of distal airways, we exposed cultures of commercially available human primary small airway epithelial cells (Lonza) to PBS or CSE. CSE exposure progressively increased the levels of LRP1 protein up to 1.5-fold in 24 h, when analyzed by Western blot (Fig. 1D), suggesting a consistent pattern in smoke-induced LRP1 expression along the respiratory tract.

A commercially available HBEC was then transfected with LRP1 siRNA or a scrambled siRNA and treated with CSE or PBS for 48 h. *LRP1* siRNA caused visible morphological changes. *LRP1*-siRNA transfected cells appeared elongated, with protrusions and filopodia, and they expanded sparsely, with visibly less cell-cell membrane contacts than scrambled-siRNA-transfected cells (Fig. 1E). This phenotype is consistent with a higher activity of Rho kinase and actin cytoskeleton, as has already been described for LRP1 knockout Schwann cells (37). In the presence of CSE, these effects were exacerbated in LRP1-deficient cells. Table 1 shows quantifications of these cellular morphometric parameters. Consistently with the visual observations, treatment with CSE significantly increased the cellular perimeter, mean radius, and maximum span of the cellular edges, while decreasing cell body density. In *LRP1*-siRNA transfected cells, CSE further increased the area covered by the minimum frame enclosing the cellular body (*P* < 0.001) and other CSE-derived effects, such as cellular perimeter, mean radius, and maximum span of cellular edges, showed increased trends. This quantification of morphologic parameters indicates increased spikiness and decreased roundness, suggesting actin cytoskeleton rearrangements.

**Table 1:** Measures of cultured cell shape in microscopy images

Parameter	Scrambled siRNA + PBS (Average ± SEM)	*LRP1* siRNA + PBS (Average ± SEM)	Scrambled siRNA + CSE (Average ± SEM)	*LRP1* siRNA + CSE (Average ± SEM)
Area (pixel^2^)	23,818 ± 598	22,481 ± 775.6	49,639 ± 3,229∗∗∗∗	59,907 ± 2,640∗∗∗∗^,#^
Perimeter (pixel)	617.5 ± 8	605 ± 13	896 ± 33∗∗∗	967 ± 22∗∗∗
Maximum span (pixel)	251 ± 4	248 ± 6	369 ± 16∗∗∗	389 ± 10∗∗∗
Mean radius (pixel)	101 ± 2	99 ± 2	150 ± 6∗∗∗	160 ± 4∗∗∗
Density (pixel/pixel^2^)	0.93 ± 0.004	0.93 ± 0.007	0.86 ± 0.01∗∗∗	0.88 ± 0.01∗∗∗^,###^

PBS versus smoke treatments: ∗*P* < 0.05, ∗∗*P* < 0.005, ∗∗∗*P* < 0.001, and ∗∗∗∗*P* < 0.0001.

Scrambled versus *LRP1* siRNA: ^#^*P* < 0.05, ^##^*P* < 0.005, ^###^*P* < 0.001, and ^####^*P* < 0.0001.

LRP1 is required for skin cell migration during wound healing (38), and because airway re-epithelization follows a similar process, we tested whether LPR1 deficiency affected adhesion and migration abilities. *LRP1*-siRNA transfected cells had significantly decreased migration (0.7-fold, *P* < 0.05) through a collagen-coated transwell. This decreased migration continued after the cells were pretreated with CSE as HBEC migration decreased ∼50% both for scrambled-siRNA and *LRP1*-siRNA transfected cells (*P* < 0.01; Fig. 1F) suggesting defective repair functions for *LRP1*-siRNA transfected cells both at baseline and after CSE. Cellular adhesion was not impacted by LRP1 loss, but pretreatment of the cells with CSE increased adhesion to the collagen matrix (Fig. 1F). Since TGF-β promotes reorganization of the cytoskeleton and LRP1 can sequester TGF-β (39), we next tested the cultures for SMAD/TGF-β pathway activation. Both in the presence of PBS or CSE, *LRP1*-siRNA transfected cells showed higher SMAD/TGF-β transcriptional activity than scrambled-siRNA transfected cells (*P* < 0.05; Fig. 1F). These data suggested that airway epithelial cells require LRP1 to limit the damaging effects of smoke.

Repeated exposure to smoke’s toxicants causes COPD and other chronic lung diseases. Patients with COPD showed decreased number of airway epithelial club cells (40). Club cells detoxify inhaled toxicants and are progenitor cells for reepithelization after injury. In mice, depletion of club cells caused peribronchiolar fibrosis (41), a remodeling process common to multiple chronic lung injuries. Therefore, we focused on club cells for our subsequent animal studies.

### Club cell-specific LRP1 deletion in mice

**Fig. 2:**
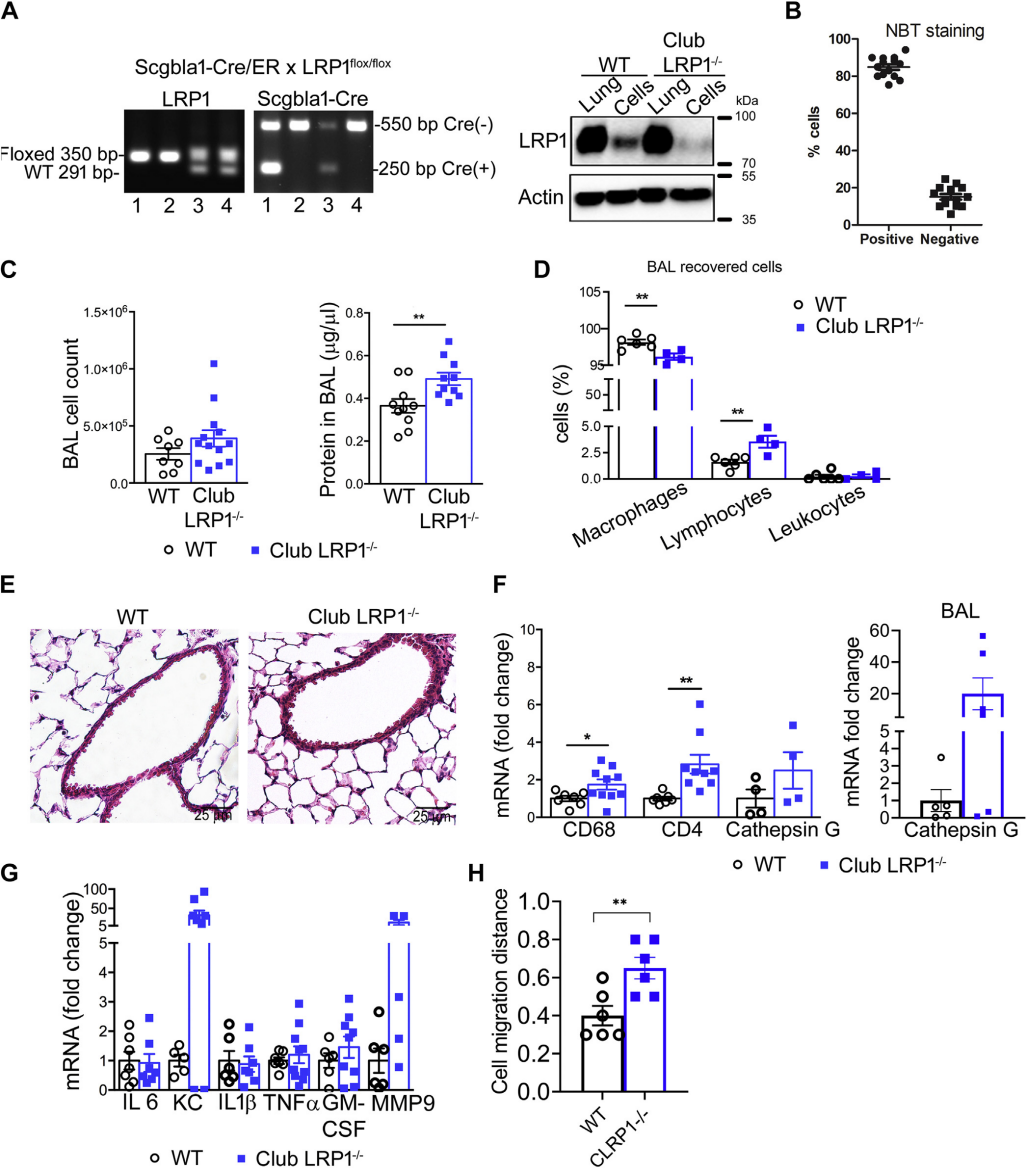
LRP1 deletion in club cells caused whole lung inflammation. A: Genotyping strategy for detection of floxed *Lrp1* and Cre presence by PCR. Lane 1: club-*Lrp1*^−/−^, lane 2: WT, lane 3: hemizygous for *Lrp1*^flox^ and Cre, lane 4: hemizygous for *Lrp1*^flox^ and Western blot for LRP1 in whole lung homogenates and isolated club cells from WT and club *Lrp1*^−/−^ mice. B: Confirmation of club cells purity in the isolated fraction. C: Cell number and protein concentration in BAL fluid. D: Cell types recovered in the BAL fluid. E: H&E staining. F: whole lung and BAL-cell qPCR for markers of lung inflammatory cells. *Cd68* serves as marker of macrophages, *Cd4* as marker of lymphocytes, and cathepsin G as marker of neutrophils. G: qPCR for inflammatory cytokines in whole lung lysates. H: Under-agarose distance migrated by bone marrow-derived cells toward lungs from WT or club *Lrp1*^−/−^ mice. ∗*P* < 0.05, ∗∗*P* < 0.01.  WT;  club *Lrp1*^−/−^.

Because club cells are abundant in human terminal airways and mouse airways of different diameters (42), we generated tamoxifen-inducible club cell-specific LRP1 knockout mice (club *Lrp1*^−/−^) using the secretoglobin 1A1 promoter. LRP1 deletion was induced after complete lung maturation and confirmed by Western blot for LRP1 after isolation of airway epithelial cells as in the study by Oreffo *et al.* (22) (Fig. 2A). This method rendered a cell suspension where >85% of the cells were nitroblue tetrazolium-positive, confirming the club-cell enrichment (Fig. 2B). Isolated cells showed a ∼95% reduction in LRP1 protein, whereas whole lung homogenates did not show differences between WT and club *Lrp1*^−/−^, likely because of other LRP1-expressing cells in the lung. There was no knockout of LRP1 in other tissues known to express high levels of LRP1, such as liver or muscle (supplemental Fig. S1), confirming the specificity of the deletion. A mild decrease of LRP1 protein was observed in livers of some club *Lrp1*^−/−^ mice after smoke exposures. The transcriptional regulation of LRP1 is cell type specific (43, 44, 45), and the airway inflammation encountered (described later) may impact homeostasis in extrapulmonary tissues (46).

The phenotype of club *Lrp1*^−/−^ mice was evaluated at 12 weeks of age, 5–6 weeks after tamoxifen injections. Club *Lrp1*^−/−^ mice showed increased absolute cell number and protein in their BAL (Fig. 2C; *P* < 0.01). The cells recovered were mostly macrophages, with a small contribution of lymphocytes and polymorphonuclear leukocytes. The proportion of lymphocytes increased from 1.6% to 3.5% of the cells recovered. Because the proportion of macrophages in BAL decreased by the same extent as the increase in lymphocytes (Fig. 2D), the overall effect was a marked increase in macrophage number.

H&E staining of lung sections did not show gross morphological differences between WT and club *Lrp1*^−/−^ mice. Airways showed only mildly thickened epithelium, and subepithelial immune infiltrates were observed in some areas in club *Lrp1*^−/−^ mice (Fig. 2E). Marker proteins for immune cells and inflammatory genes were analyzed in lung homogenates after removal of BAL (Fig. 2F, G). The pan-macrophage *Cd68* showed 1.7-fold more mRNA in club *Lrp1*^−/−^ than in WT mice (*P* < 0.05; Fig. 2F), whereas lymphocyte-specific *Cd4* mRNA expression was 2.8-fold greater (*P* < 0.001) than in WT. Keratinocyte chemoattractant (*KC*) and *MMP-9* showed a trend toward increased levels in club *Lrp1*^−/−^ mice (Fig. 2G). KC has neutrophil-chemotactic properties, and consistently, neutrophil-specific cathepsin G showed a trend toward increased expression both in lung tissue and in BAL cell pellet (Fig. 2F). Therefore, lung parenchyma and bronchoalveolar space of club *Lrp1*^−/−^ mice had infiltration of inflammatory cells and baseline inflammation. The ability of lungs of club *Lrp1*^−/−^ mice to recruit inflammatory cells was confirmed in an underagarose migration test. Bone marrow-derived immune cells migrated ∼1.5-fold further toward lung explants from club *Lrp1*^−/−^ mice than toward those from WT mice (Fig. 2H; *P* < 0.01). The overall proinflammatory profile was not exacerbated by aging. Mice aged to 8 months showed no significant differences in BAL protein, proinflammatory cytokines, or immune cell markers in lung tissue (supplemental Fig. S2), suggesting there was no significantly sustained inflammation.

### Pulmonary function testing and histology of club *Lrp1*^−/−^ mice

**Fig. 3:**
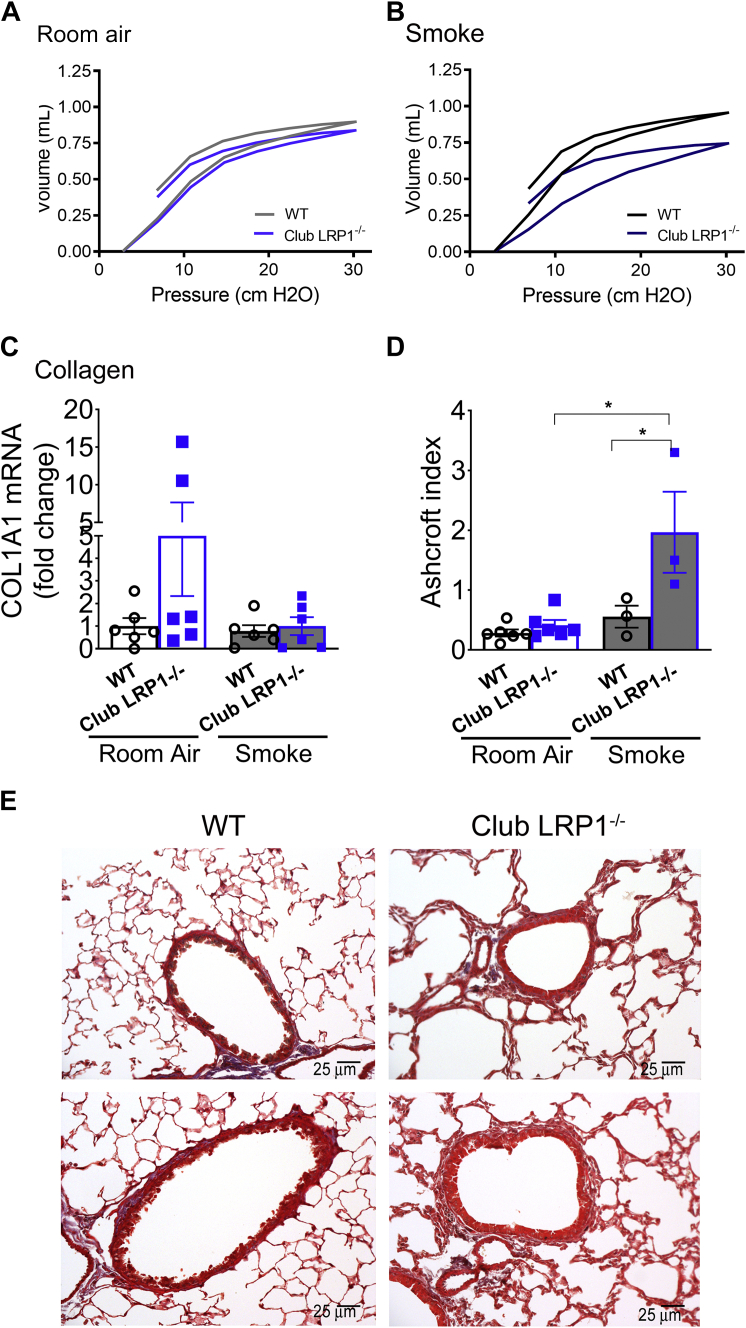
Pulmonary function and histology in WT and club *Lrp1*^−/−^ mice. Mice were exposed to smoke or room air for 6 months. A: Pressure-volume loops for room air-exposed mice (N = 5–13/condition). B: Pressure-volume loops for smoke-exposed mice. C: Collagen mRNA expression in lung homogenates of WT and club *Lrp1*^−/−^ mice after room air or smoke exposure. D: Ashcroft index in WT and club *Lrp1*^−/−^ mice after room air or smoke exposure. E: Representative images of Trichrome staining for male mice. Statistics: two-way ANOVA followed by Bonferroni post-test. ∗*P* < 0.05, ∗∗*P* < 0.01.  WT;  club LRP1^−/−^.

We assessed whether club *Lrp1*^−/−^ mice had alterations in lung function or pathology on room air. Eight-month-old club *Lrp1*^−/−^ mice had a slight shift of the pressure-volume loop to the right and down when compared with their WT littermates (Fig. 3A), which was consistent with a trend toward decreased inspiratory capacity (IC) and compliance (Cst) that did not reach significance on room air conditions (supplemental Table S1). On average, we noticed a greater body weight gain in male mice for club *Lrp1*^−/−^ than for WT mice (supplemental Fig. S3), but such a change would explain their slightly larger pulmonary volumes (supplemental Table S2).

We then challenged 2-month-old mice with 6 months of second-hand smoke as a COPD trigger. Smoke exposure dramatically shifted the pressure-volume loop right and down in club *Lrp1*^−/−^ mice, whereas WT mice showed a subtle trend toward a shift to the left and up, as expected (47, 48) (Fig. 3B). While a left and up shift suggests obstructive disease, a right and down shift indicates restrictive disease and reduced pulmonary function associated with increased pressure needs for maximum inflation. Indeed, compliance in WT mice after smoke exposure was 0.09 ± 0.005 ml/cmH_2_O, whereas in club *Lrp1*^−/−^, it was decreased to 0.07 ± 0.02 ml/cmH_2_O (*P* < 0.01; supplemental Table S1). IC was 1.03 ± 0.05 ml in WT and only 0.79 ± 0.20 ml in club *Lrp1*^−/−^ mice (*P* < 0.01; average ± SD; supplemental Table S1). This suggested that the lungs in club *Lrp1*^−/−^ mice were stiffer and potentially fibrotic. In addition, airway resistance (Rn and Rrs) was increased in club *Lrp1*^−/−^ mice after smoke exposure supplemental Table S1), suggesting coexistence of restrictive disease (decreased IC and Cst) and obstructive disease (decreased forced expiratory volume_0.05_/forced vital capacity). Supplemental Fig. S3 and supplemental Tables S2 and S3 show that these data segregated by sex. Overall, males and females showed comparable phenotypes.

We assessed the ECM in WT and club *Lrp1*^−/−^ mice. Collagen 1A1 mRNA expression in lung homogenates trended toward an increase in club *Lrp1*^−/−^ mice (Fig. 3C). In lung sections stained for collagen, club *Lrp1*^−/−^ mice showed slightly more collagen deposition and subepithelial immune cell infiltrates than WT mice before smoke (Fig. 3D, E). After smoke, both WT and club *Lrp1*^−/−^ mice showed thickened airway walls, increased deposition of collagen, and areas of epithelial denudation. The Ashcroft score showed a greater degree of fibrosis induction in club *Lrp1*^−/−^ (*P* < 0.05; Fig. 3D). These data suggest that loss of airway epithelial LRP1 enhances susceptibility to smoke-induced airway remodeling and pulmonary fibrosis. Supplemental Fig. S4 show that these data segregated by sex.

### Proteomic characterization of primary club cells

**Fig. 4:**
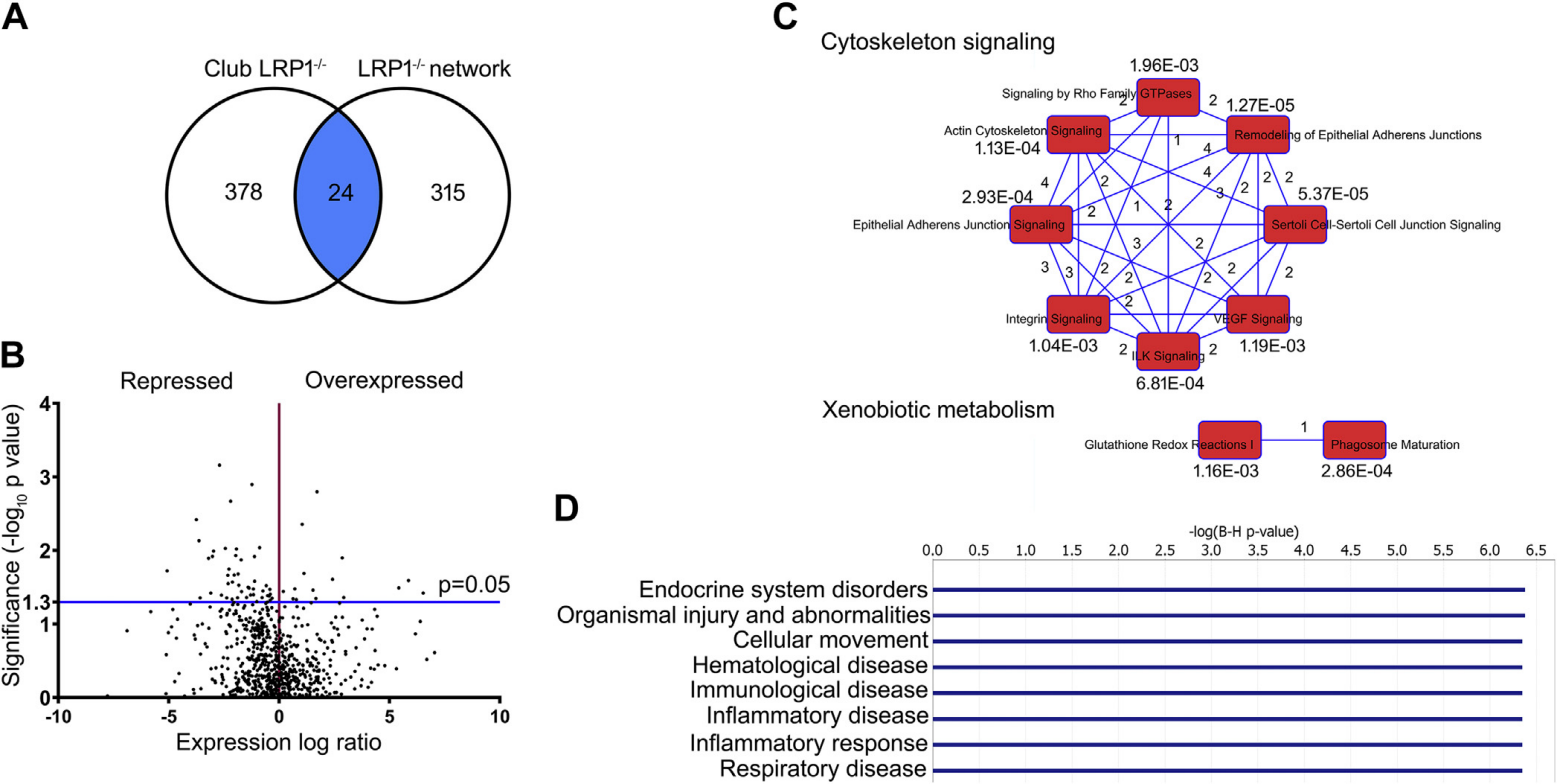
Untargeted proteomics and pathway analysis of isolated club cells from WT and club *Lrp1*^−/−^ mice. A: Overlap between the club cell proteome in club-LRP1^−/−^ mice and known LRP1-interacting proteins (LRP1 network). B: Volcano plot of proteins detected in club cells. Expression levels are relative to WT mice. Significantly overexpressed proteins in club *Lrp1*^−/−^ mice occupy the upper right quadrant and significantly underexpressed proteins occupy the upper left quadrant. Proteins in the lower part of the graph correspond with nonstatistically significant trends. C: Top 10 canonical pathways that grouped the proteins with significant expression changes in club *Lrp1*^−/−^ mice. Number of proteins common to each pair of pathways and *P* value of overlap with the canonical pathway are shown. D: Most significant diseases involving the proteins with significant expression changes in club *Lrp1*^−/−^ mice. *P* value of overlap with canonical disease pathway is shown.

To determine why LRP1 loss increased baseline inflammation and worsened disease after smoke exposure, we performed a proteomic analysis (Bioproximity, LLC, VA) in primary cells isolated from room air-exposed and smoke-exposed mice. The complete dataset for this experiment has been deposited in the public repository MassIVE, with accession number MSV000083163 (ftp://massive.ucsd.edu/MSV000083163). IPA software was used for bioinformatic analysis. We first interrogated IPA for the proteins already known to interact with LRP1 (“LRP1 network”). Figure 4A shows a Venn diagram representing the overlap between LRP1 network and the proteome of club cells isolated from club *Lrp1*^−/−^ mice. Cells from club *Lrp1*^−/−^ mice showed 378 different proteins that were not part of the described LRP1 network, 24 proteins that overlapped with known members of LRP1 network, and 315 proteins that belong to LRP1 network but were not identified in our club cell sample and therefore unlikely to participate in the phenotype of club *Lrp1*^−/−^ mice. The identities of each of these proteins are detailed in supplemental Tables S4–S6.

Figure 4B shows all differentially expressed proteins in a volcano plot. The ID for all the plotted proteins, their fold change, and *P* values are detailed in supplemental Table S7. Consistent with the findings in lung homogenates, proteins that participate in inflammatory processes were increased in club *Lrp1*^−/−^ mice: cathepsin B and HSP70 member 5 were significantly increased (*P* < 0.05; supplemental Table S7), and other cathepsins showed trending increases (not significant [ns], supplemental Table S7). Cytokines KC and MMP-9 were not detected in club cells, suggesting that macrophages and not club epithelial cells were the source of these cytokines detected earlier in whole lung homogenates by qPCR in Fig. 2F.

Proteins were then grouped in metabolic and signaling pathways (Fig. 4C). The pathways with greatest representation and best match comprised two major categories: xenobiotic metabolism and cytoskeleton signaling. In addition, the differentially expressed proteins grouped with high significance in disease categories that included organismal injury, inflammatory response, and respiratory disease (Fig. 4D). These results prompted us to further analyze the role of LRP1 in xenobiotic metabolism and cytoskeleton signaling separately.

### Club *Lrp1* deletion altered interactions between cytoskeleton and ECM

**Fig. 5:**
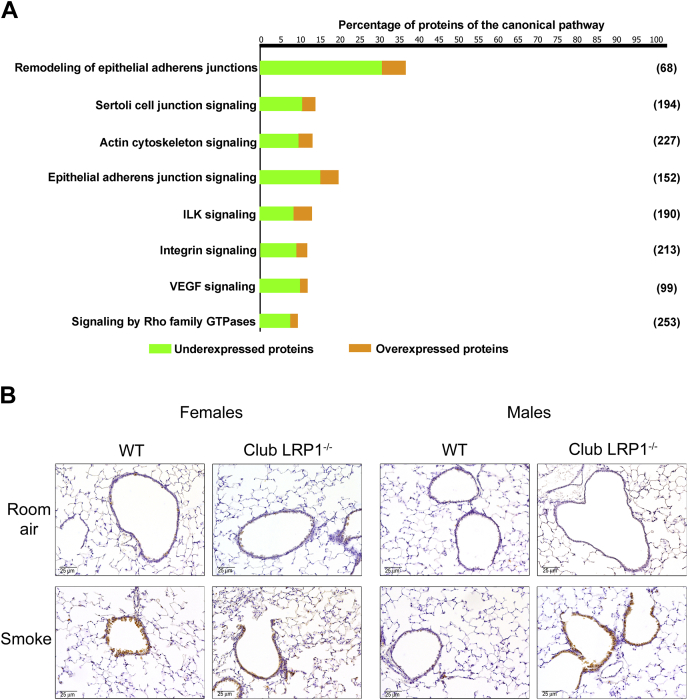
Analysis of cytoskeleton signaling in WT and LRP1-deficient airway cells. A: Cellular pathways involved in cytoskeleton signaling. Overexpressed (orange) and repressed (green) proteins in club *Lrp1*^−/−^ for each pathway are shown. Only proteins with expression changes with *P* < 0.05 were considered for this analysis. Percentage of detected proteins relative to the total number of proteins comprising the pathway is indicated in the axis, and absolute number of proteins (100% of pathway) is indicated in brackets for each pathway. B: Ki67 staining for cellular proliferation in lung sections. Representative images from female and male mice are shown.

Changes in cytoskeleton signaling pathways in LRP1-deleted club cells were consistent with our in vitro data (Figs. 1 and 5A). These pathways maintain cellular morphology and mediate cellular interactions with the ECM by regulating adhesion and migration processes. Proteins involved in cell contractility and adhesion, including myosin heavy chain 10 (1.15-fold) or beta-actin (4-fold) exhibited trends for increases in cells from club *Lrp1*^−/−^ mice (Table 2 and supplemental Table S7). Conversely, cytoskeletal proteins that form intermediate filaments or participate in cell locomotion decreased. That was the case of actinin alpha 1, spectrin alpha (*P* < 0.05; Table 2), and myosin light chains 9 and 12B (ns, supplemental Table S7). Most pathways showed a majority of repressed proteins. While many individual proteins showed trends, the pathway analysis differences reached statistical significance (Fig. 5A). The canonical pathways involved in this category and the proportion of underexpressed and overexpressed proteins are shown in Fig. 5A. Smoke exposure caused in these pathways a specific proteome fingerprint that did not follow the same trends as in room air conditions but showed that club *Lrp1*^−/−^ mice had predicted increases in signaling by actin cytoskeleton, integrins, and Rho-GTPases in club *Lrp1*^−/−^ mice (supplemental Fig. S5). All the proteins identified in the mice subjected to room air and smoke exposures, with their expression levels and *P* values are detailed in supplemental Table S7.

**Table 2:** Proteins involved in cytoskeleton metabolism

Symbol	Entrez Gene Name	UniProt ID	Expression Log Ratio	Expression *P* Value
ACTA1	actin alpha 1, skeletal muscle	Q61272	−1.831	5.68E-01
ACTA2	actin alpha 2, smooth muscle	P62737	−1.511	6.01E-01
ACTB	actin beta	E9Q2D1	2.03	5.60E-01
ACTC1	actin alpha cardiac muscle 1	P68033	−1.168	6.00E-01
ACTG1	actin gamma 1	B1ATY1	−3.125	5.86E-02
ACTG2	actin gamma 2, smooth muscle	P63268	−1.557	4.73E-01
ACTN1	actinin alpha 1	Q7TPR4	−1.159	3.98E-02
ACTN4	actinin alpha 4	Q8BP35	−2.029	2.15E-02
ACTR3	actin related protein 3	Q9ERF8	−0.58	3.01E-02
ARF4	ADP ribosylation factor 4	E9Q798	−0.734	3.54E-02
FLNA	filamin A	Q3U7N9	−3.58	4.40E-02
GNAI2	G protein subunit alpha i2	A0A0A6YWA9	−1.881	4.38E-02
IQGAP1	IQ motif containing GTPase activating protein 1	Q9JKF1	−2.37	4.41E-02
MYH10	myosin heavy chain 10	Q4KMN4	0.187	1.41E-01
SPTAN1	spectrin alpha, non-erythrocytic 1	B7ZWK3	−1.717	1.34E-02
SPTB	spectrin beta, erythrocytic	E9Q397	1.125	2.05E-02
SPTBN1	spectrin beta, non-erythrocytic 1	A0A0A0MQG2	−2.971	1.02E-02
TUBA1B	tubulin alpha 1b	O89052	−3.344	1.85E-01
TUBA1C	tubulin alpha 1c	Q3TIZ0	−1.393	2.20E-01
TUBA4A	tubulin alpha 4a	A0A087WRB4	−0.984	2.14E-01
TUBA8	tubulin alpha 8	Q9JJZ2	−2.324	4.05E-01
TUBB	tubulin beta class I	Q80ZV2	−0.432	2.65E-01
TUBB1	tubulin beta 1 class VI	A2AQ07	6.691	3.03E-01
TUBB3	tubulin beta 3 class III	Q9CRT0	−1.867	5.39E-02
TUBB6	tubulin beta 6 class V	Q3U9U3	0.262	5.81E-02
VIM	vimentin	Q3UAX1	−1.475	4.35E-02
YWHAE	tyrosine 3-monooxygenase/tryptophan 5-monooxygenase activation protein epsilon	Q3V453	−1.116	1.21E-02

Also in agreement with the in vitro studies, club *Lrp1*^−/−^ mice showed increased TGF-β signaling and cellular staining of the proliferation marker Ki67 after smoke exposure (Fig. 5B).

### Club *Lrp1*^−/−^ decreased oxidative stress quenching potential

**Fig. 6:**
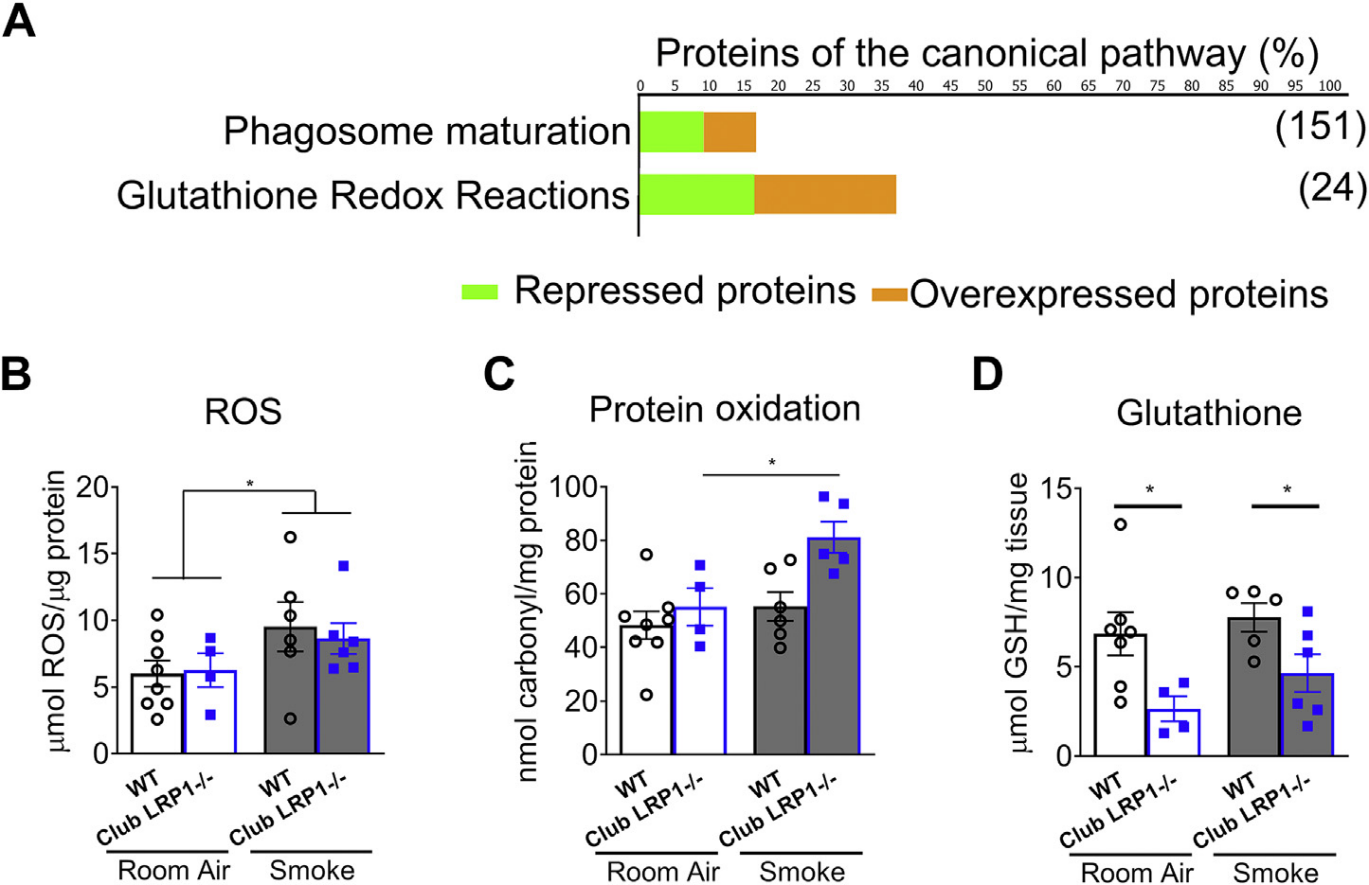
Analysis of xenobiotic metabolism in WT and club *Lrp1*^−/−^ mice. A: Cellular pathways involved in xenobiotic metabolism. Overexpressed (orange) and repressed (green) proteins in club *Lrp1*^−/−^ for each pathway are shown. Only proteins with expression changes with *P* < 0.05 were considered for this analysis. Percentage of detected proteins relative to the total number of proteins comprising the pathway is indicated in the axis, and absolute number of proteins (100% of pathway) is indicated in brackets for each pathway. B: ROS in whole lungs from WT and club *Lrp1*^−/−^ mice, before and after smoke exposure. C: Permanent protein oxidation in whole lungs from WT and club *Lrp1*^−/−^ mice before and after smoke exposure. D: Total glutathione in whole lungs from WT and club *Lrp1*^−/−^ mice before and after smoke exposure. Statistics: two-way ANOVA followed by Bonferroni post-test for C and D. ∗*P* < 0.05.  WT;  club *Lrp1*^−/−^.

Our data thus far suggest that LRP1 is involved in the normal response to inflammation. Differentially expressed proteins involved in xenobiotic metabolism are represented in Fig. 6A and supplemental Fig. S6 and detailed in Table 3. Glutathione-disulfide reductase, some isoforms of glutathione-*S*-transferase, and detoxifying enzymes like peroxiredoxins (peroxiredoxins 4, 5, and 6) showed drastically decreased levels (*P* < 0.05 for all, 1.4–5-fold change). Other detoxifying enzymes like glutathione peroxidase 1 or peroxiredoxins 1–3 showed trends toward increased levels (ns, 1.8–8.5-fold change). Catalase and cytochrome p450 oxidoreductase, which can generate ROS, showed a trending increase in club *Lrp1*^−/−^ mice (ns, 1.7-fold and 9.5-fold change, respectively). These proteins are grouped in glutathione redox reactions and phagosome maturation pathways (Fig. 6 and Table 3) for mice exposed to room air. Epithelial cells are capable of phagocytosis (49). The phagosome maturation has multiple steps in common with endosome trafficking, and it is a source of ROS as a mechanism for detoxification (50, 51, 52). Consistent with this, smoke exposure increased proteins involved in glutathione and xenobiotic signaling pathways (supplemental Fig. S6).

**Table 3:** Proteins involved in xenobiotic and glutathione metabolism

Symbol	Entrez Gene Name	UniProt ID	Expression Log Ratio	Expression *P* Value
ADH5	alcohol dehydrogenase 5 (class III), chi polypeptide	Q6P5I3	−1.825	2.97E-02
Aldh1a7	aldehyde dehydrogenase family 1, subfamily A7	O35945	−2.197	2.14E-03
CAT	catalase	Q3UF58	0.8	3.10E-01
CES1	carboxylesterase 1	Q8VCT4	−1.226	1.27E-03
GLO1	glyoxalase I	Q9CPU0	−0.881	9.14E-03
GPX1	glutathione peroxidase 1	A0A0A6YY34	0.921	9.29E-01
GSR	glutathione-disulfide reductase	Q3UAS7	−2.116	2.69E-02
GSTA3	glutathione S-transferase alpha 3	K9JA27	−2.376	1.35E-01
Gsta4	glutathione S-transferase, alpha 4	P24472	3.49	2.10E-01
GSTA5	glutathione S-transferase alpha 5	Q6P8Q0	−0.109	6.95E-01
GSTK1	glutathione S-transferase kappa 1	Q9DCM2	−4.28	9.50E-01
GSTM1	glutathione S-transferase mu 1	D3YX76	0.678	5.78E-01
GSTM2	glutathione S-transferase mu 2	D3YVP6	1.665	5.87E-01
Gstm3	glutathione S-transferase, mu 3	P19639	−0.006	6.19E-01
GSTM5	glutathione S-transferase mu 5	P10649	−0.749	5.41E-01
GSTO1	glutathione S-transferase omega 1	O09131	−1.111	2.61E-02
GSTP1	glutathione S-transferase pi 1	P19157	−1.269	1.24E-01
Gstt1	glutathione S-transferase, theta 1	Q9DCY6	4.146	3.97E-01
IDH1	isocitrate dehydrogenase (NADP(+)) 1	Q3TJ51	−0.518	4.11E-02
MGST1	microsomal glutathione S-transferase 1	E9QJW0	2.064	1.75E-01
MGST1	microsomal glutathione S-transferase 1	E9QJW0	2.064	1.75E-01
PDIA3	protein disulfide isomerase family A member 3	P27773	−0.329	6.00E-02
PEBP1	phosphatidylethanolamine binding protein 1	Q3TGC5	−2.611	4.04E-02
PGD	phosphogluconate dehydrogenase	Q91V28	−2.434	9.33E-03
POR	cytochrome p450 oxidoreductase	E9Q997	3.253	5.25E-01
PRDX1	peroxiredoxin 1	B1AXW4	3.153	1.10E-01
PRDX2	peroxiredoxin 2	Q61171	0.611	4.49E-01
PRDX3	peroxiredoxin 3	P20108	0.325	3.36E-01
PRDX4	peroxiredoxin 4	B1AZS9	−2.362	7.50E-02
PRDX5	peroxiredoxin 5	G3UZJ4	−0.77	4.37E-02
PRDX6	peroxiredoxin 6	D3Z0Y2	−0.403	4.76E-02
PRDX6	peroxiredoxin 6	D3Z0Y2	−0.403	4.76E-02
Prdx6b	peroxiredoxin 6B	Q8BG37	2.319	3.70E-01
SOD2	superoxide dismutase 2	Q3TJA2	−0.2	9.80E-01

We assessed biochemically the oxidative damage in lung before and after the smoke challenge. Smoke exposure tended to increase ROS for lungs of both WT and club *Lrp1*^−/−^ mice (Fig. 6B). However, only club *Lrp1*^−/−^ and not WT mice had significantly increased protein oxidative damage after smoke exposure: permanent carbonyl protein modification augmented from 55.1 ± 7 nmol carbonyls/μg protein in club *Lrp1*^−/−^ room air-exposed mice to 81.1 ± 6 nmol carbonyls/μg protein after smoke (*P* < 0.05; Fig. 6C). We then measured the levels of GSH because this is the primary mechanism for detoxification of oxidant compounds used by the cells to prevent structural damage. Lungs from club *Lrp1*^−/−^ mice had decreased GSH availability both at room air and after smoke exposure. At room air conditions, total GSH mass was 6.8 ± 1 μmol GSH/mg tissue in WT mice, but only 2.6 ± 1 μmol GSH/mg tissue in club *Lrp1*^−/−^ mice; after smoke exposure, WT mice had 7.8 ± 1 μmol GSH/mg tissue, but club *Lrp1*^−/−^ mice had only 4.6 ± 1 μmol GSH/mg tissue (*P* < 0.05; Fig. 6D). These data suggest that loss of LPR1 in club cells results in GSH depletion and subsequent increased susceptibility to oxidative damage by smoke exposure. Supplemental Fig. S7 shows the data for mice segregated by sex. Excepting in ROS levels after smoke exposure, there were no sex-dependent significant differences, and both sexes followed similar trends.

Despite club *Lrp1*^−/−^ mice initially having lower levels of most proteins involved in detoxification pathways at room air conditions (Fig. 6A), these differences disappeared after completion of the smoke exposure time (supplemental Fig. S6). Smoke increased the levels of proteins involved in glutathione reactions and xenobiotic signaling in club cells from WT and club *Lrp1*^−/−^ mice. However, lung GSH remained lower in club *Lrp1*^−/−^ compared with WT mice after smoke exposure (Fig. 6C, D) despite no reduction in the levels of detoxifying enzymes, suggesting a role for LRP1 signaling in quenching the smoke-induced protein oxidative damage.

## Discussion

LRP1 performs signaling and metabolic functions in tissue and cell specific-manners, and its function in club cells is unknown. Our studies sought to understand why some SNPs and mutations in LRP1 correlate with decreased lung function in smokers (6, 9). We found that patients with COPD had increased LRP1 in the airway epithelium and hypothesized that this could serve a defense mechanism similar to its reported actions in experimental ischemic heart failure in mice (4). Club cells are abundant in human terminal bronchioles, and they function as detoxifying hubs for noxious agents and as progenitor cells for epithelial repair after injury (53). The epithelium of the small airways is a main site of action during the initiation of the inflammatory response and the onset of chronic damage (54).

**Fig. 7:**
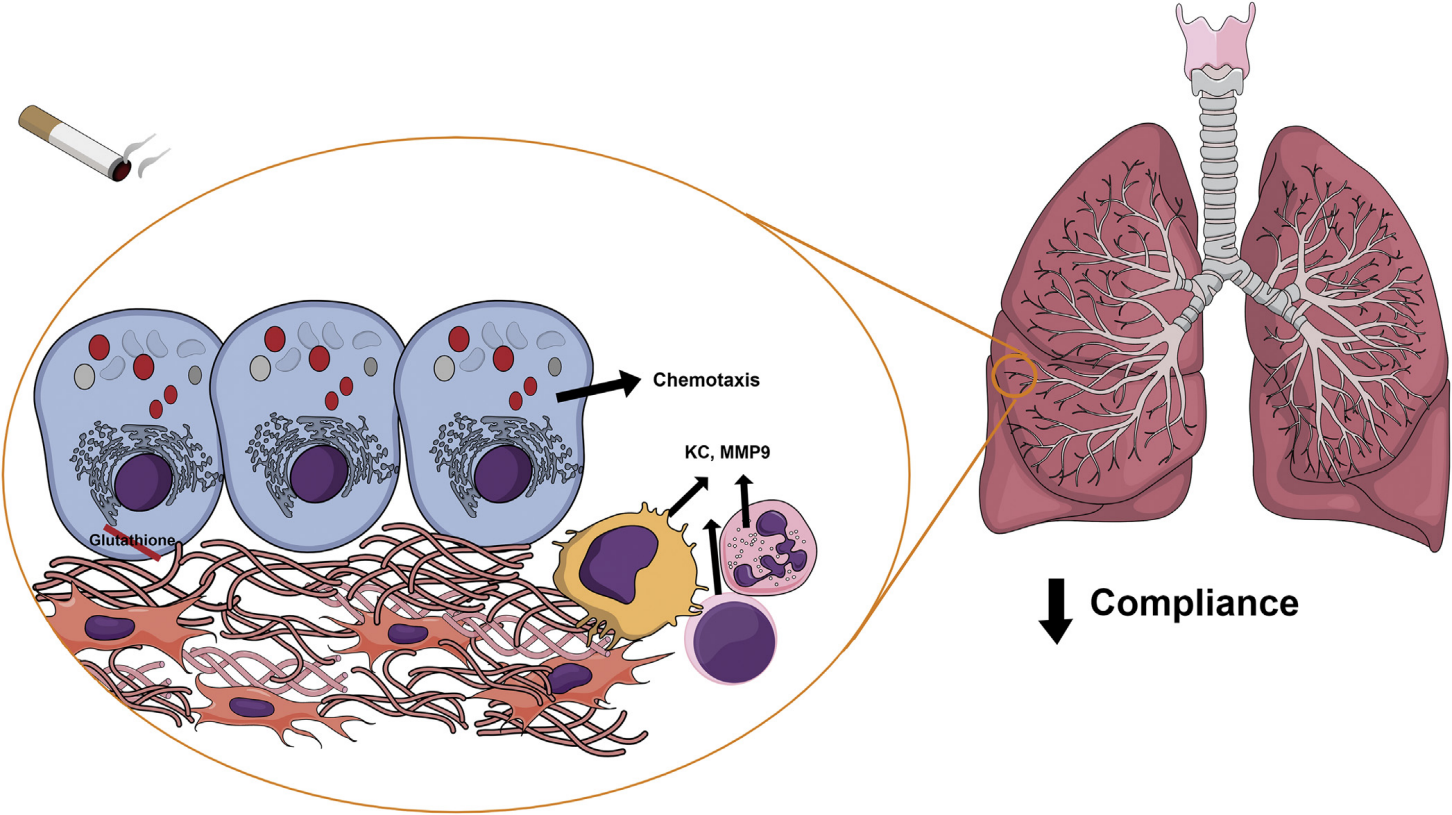
Schematic representation of the phenotype in club *Lrp1*^−/−^ mice. LRP1 deletion in club cells results in glutathione depletion, protein oxidation, and recruitment of immune cells capable of secreting proinflammatory mediators at baseline. After smoke exposure, this environment promotes increased collagen expression and extracellular matrix deposition, decreasing lung compliance. (The figure is created in the “Mind the Graph” platform).

Our in vitro data with LRP1 knockdown supported the hypothesis that club expression of LRP1 protects the lung. To ensure that tamoxifen-inducible club cell-specific *Lrp1*^−/−^ mice had normal lung development, we induced loss of LRP1 only in adulthood. Using this model, we learnt that LRP1 loss of function in club cells *1*) increased lung immune cell infiltration and inflammation, *2*) decreased pulmonary compliance, *3*) increased fibrotic scoring after smoke, *4*) dysregulated cytoskeleton signaling, *5*) increased cellular proliferation, and *6*) increased smoke-associated oxidative damage. Figure 7 depicts a schematic interpretation of the entire phenotype in the small airway niche, where loss of LRP1 in epithelial club cells results in glutathione depletion and chemotactic attraction of immune cells, which in turn can secrete further proinflammatory cytokines that prime the environment for increased oxidative damage, fibroblast collagen synthesis, and subsequent loss of pulmonary compliance after smoke exposure.

Cigarette smoke alters the airway epithelium and induces airflow obstruction in susceptible smokers. Animal models of COPD seldom recapitulate the complexity of the human disease in both airways and alveoli. Here, the loss of LRP1 expression specifically in airway epithelial club cells worsened airway diseases in smoke-exposed mice, as determined by the reduction in forced expiratory volume_0.05_/forced vital capacity. Club *Lrp1*^−/−^ mice also exhibited restrictive physiologic alterations, with marked reductions in compliance and IC after smoke exposure. This physiology is attributable to LRP1 expression in club cells, as other pulmonary cells that preserved LRP1 expression (Figs. 1 and 2) showed no compensatory effects in club *Lrp1*^−/−^ mice. Chronic smoke triggers both restrictive and obstructive changes in the lung of patients with combined pulmonary fibrosis and emphysema (CPFE) (55). It remains uncertain why the same stimulus could induce such differing physiologies within the same lung. This is an important question since CPFE individuals suffer higher rates of mortality (56), pulmonary hypertension (57), and lung cancer (58) than other patients with COPD. Given the findings from the club *Lrp1*^−/−^ mice, it is conceivable that deficient LRP1 expression in the airways could set the environment for the development of CPFE.

Although originally described as a lipoprotein receptor, LRP1 has subsequently been shown to affect a number of processes that are likely related to the development of inflammation and pulmonary disease. LRP1 can prevent activation of TGF-β receptor (II) by sequestering TGF-β or TGF-β receptor (I) (39), and in vitro, LRP1 binding to TGF-β mediates antiproliferative effects (39, 59). *Lrp1* deletion in vascular smooth muscle cells resulted in spontaneous development of pulmonary arterial hypertension by lifting the inhibition of the TGF-β-connective tissue growth factor axis (60, 61). LRP1 can decrease lung inflammation also in alveolar macrophages by its ability to clear environmental insults and in other immune cells by binding to α2-macroglubulin or tissue plasminogen activator (16). In mouse mammary epithelial cells, antibodies against LRP1 partially impaired phagocytosis (62). And by internalizing serine proteases and MMPs, LRP1 limits extracellular protease activity (63, 64). These roles of LRP1 as an anti-inflammatory receptor might explain the inflammatory phenotype of the club *Lrp1*^−/−^ mice.

LRP1 participates in cellular migratory processes, and the subsequent effects differ depending on disease and conditions of study (65). In cultured macrophages, activation of LRP1 induced cell migration (66), whereas in vivo, LRP1-deficient M1 macrophages more efficiently egressed from atherosclerotic plaques accelerating atherosclerosis regression (67). In Schwann cells in the nervous system, LRP1-ligand binding promoted cellular migration by activating Rho-GTPases, and inhibiting this cascade led to further adhesion in vitro (37). In vivo, LRP1 loss in Schwann cells promoted faster but abnormal nerve regeneration after injury, causing pain onset and loss of motor function (68, 69). Consistently, in our experiments, LRP1 knockdown decreased migration through a collagen matrix in vitro, and primary cells from club *Lrp1*^−/−^ mice showed multiple modifications of cytoskeleton-related and Rho-GTPase pathways. In vivo, this could impair migration and compromise airway epithelial repair after injury.

Glutathione deficiency, the other major trait associated with the proteome of club *Lrp1*^−/−^, is a characteristic of idiopathic pulmonary fibrosis, and this is associated with the ability of TGF-β to inhibit glutathione synthesis (70, 71). TGF-β also suppresses activity and mRNA of antioxidant enzymes (reviewed in Refs. (72, 73)). Reversing protein oxidation and *S*-glutathionylation was beneficial in models of lung fibrosis (74). The expression of GCLC, the rate-limiting enzyme for the synthesis of glutathione, and its activity can be finely modulated postranslationally by multiple mechanisms (reviewed in Ref. (75)). Consistent with the club *Lrp1*^−/−^ mice at baseline, GCLC^−/−^ mice have a marked decrease in glutathione availability, but no phenotype becomes evident until the mouse is challenged (76). A similar mechanism could explain why the protein oxidative damage is increased in club *Lrp1*^−/−^ mice only after smoke exposure and not at baseline. Likely, their already decreased glutathione levels are insufficient to quench the smoke exposure-originated ROS as effectively as in the WT mice. To our knowledge, this is the first study to link LRP1 to levels of antioxidant proteins.

We were surprised that the proteomic analysis did not show a significant impact in the lipid metabolic pathways. Lipidomic studies have shown reprogramming of fatty acid and ceramide metabolism in airway epithelial cells during cystic fibrosis and viral infections (77). Lipid metabolism is tightly regulated transcriptionally and postranslationally (78), and it is possible that a transcriptomic analysis would have identified lipid metabolic pathways. Proteomic analysis like the one in our study usually identifies the most abundant proteins in the sample. Since club epithelial cells are highly specialized in detoxification and repair functions, the pathways identified by our untargeted approach were consistent with their main functions. A transcriptomic analysis, which usually renders a much higher number of hits, or a targeted metabolomic analysis would likely show additional differentially regulated pathways, as it occurs in other studies that use integrative systems analysis approaches (79, 80). Also, the sensitivity of the analysis likely explains the failure to detect which cytokines in club cells could initiate the chemotaxis observed. Other pulmonary cells, such as endothelial cells, or the infiltrated immune cells could play an amplifying role and be the main source of the proinflammatory mediators detected in whole lung. MMP9, which actively participates in bronchial epithelial cell migration (81), was detectable in isolated club cells only by qPCR but not in the proteomic dataset, and its trending decrease in club *Lrp1*^−/−^ mice (supplemental Fig. S2) further supports our data on limited migration ability with loss of LRP1, as well as the role of other cell types in cytokine and MMP production.

In summary, our data explain the genetic associations of LRP1 with human disease. This protein is highly expressed in human epithelial cells, and our animal studies show that its expression in club cells affects lung pathophysiology. LRP1 in airway epithelial cells modulates the development of smoke-induced disease. Further research will help clarify the crosstalk between club cells and other pulmonary cells. However, our data suggest that activation of LRP1 is a protective mechanism that could limit the deleterious effects of known lung toxicants.

## Data availability

All presented data are contained in the supplemental tables, and the original proteomic complete dataset has been deposited in the public repository MassIVE, with accession number MSV000083163.

## Supplemental data

This article contains supplemental data.

## Conflict of interest

The authors declare that they have no conflicts of interest with the contents of this article.
