# Supplementary material for: LRP1 loss in airway epithelium exacerbates smoke-induced oxidative damage and airway remodeling
Source: J Lipid Res. 2022 Feb 21;63(4):100185. doi: 10.1016/j.jlr.2022.100185 (PMC8953659; doi:10.1016/j.jlr.2022.100185)
Supplement: Supplemental Figures S1–S7 [file mmc1.pdf]

Supplemental Figure S1.

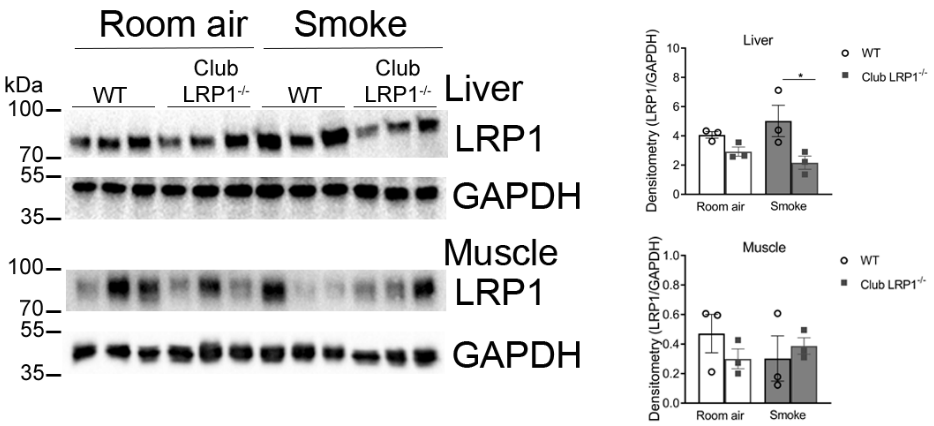

Supplemental Figure S1. LRP1 expression in club *Lrp1*$^{-/-}$ mice. Liver and muscle were collected form WT and Club LRP1-/- mice exposed to room air or smoke and western blotted against LRP1 and GAPDH to confirm lack of unspecific deletion on LRP1 in highly metabolic tissues. Western blot densitometry is shown.

Supplemental Figure S2.

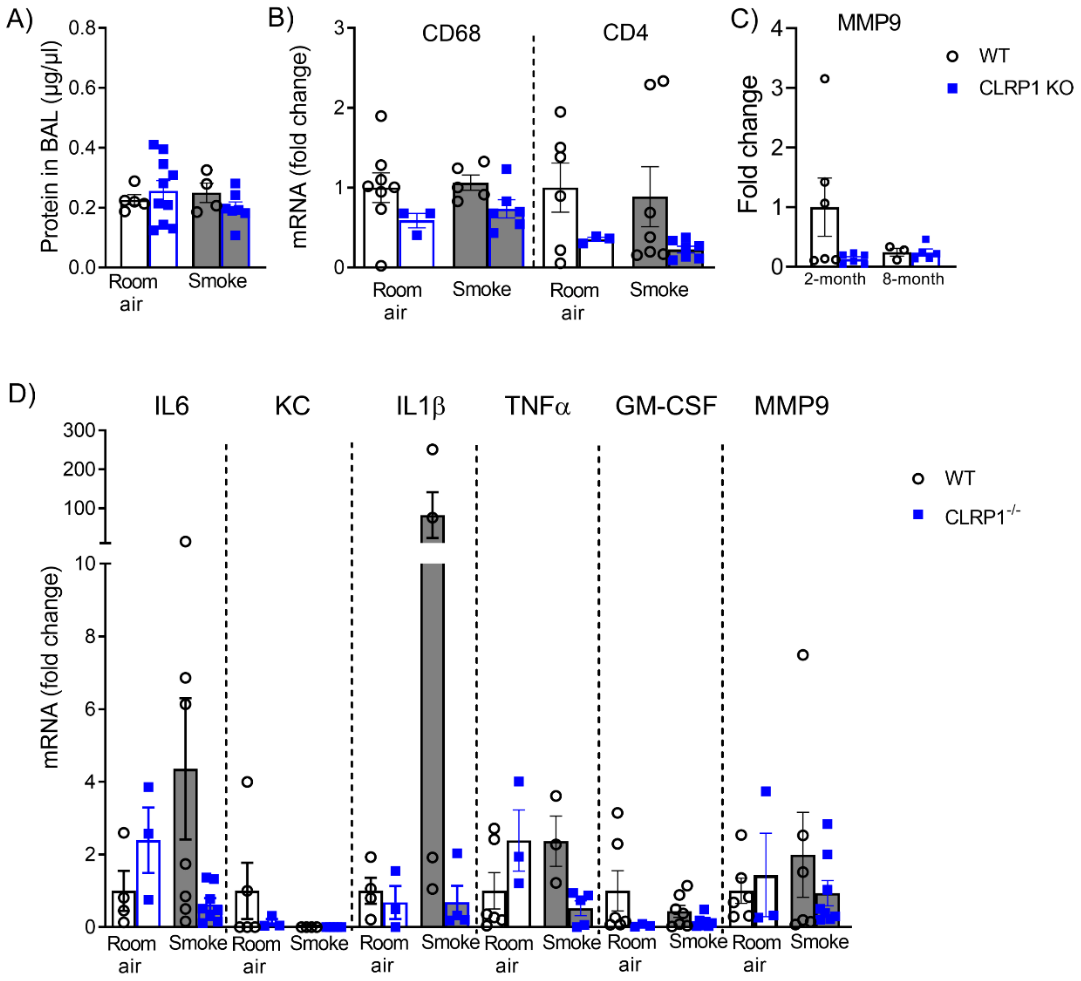

Supplemental Figure S2. Lung inflammatory profile after 6 months of room air or smoke exposure. A) protein concentration in BAL fluid from WT and club *Lrp1*$^{-/-}$ mice B) whole lung qPCR for markers of lung inflammatory cells. CD68 serves as marker for macrophages, CD4 as marker for lymphocytes, C) qPCR for MMP9 in isolated club cells from room air exposed mice at 2 and 8 months of age D) qPCR for inflammatory cytokines in whole lung lysates.

*p<0.05, **p<0.01. ○ WT ■ club *Lrp1*$^{-/-}$

Supplemental Figure S3.

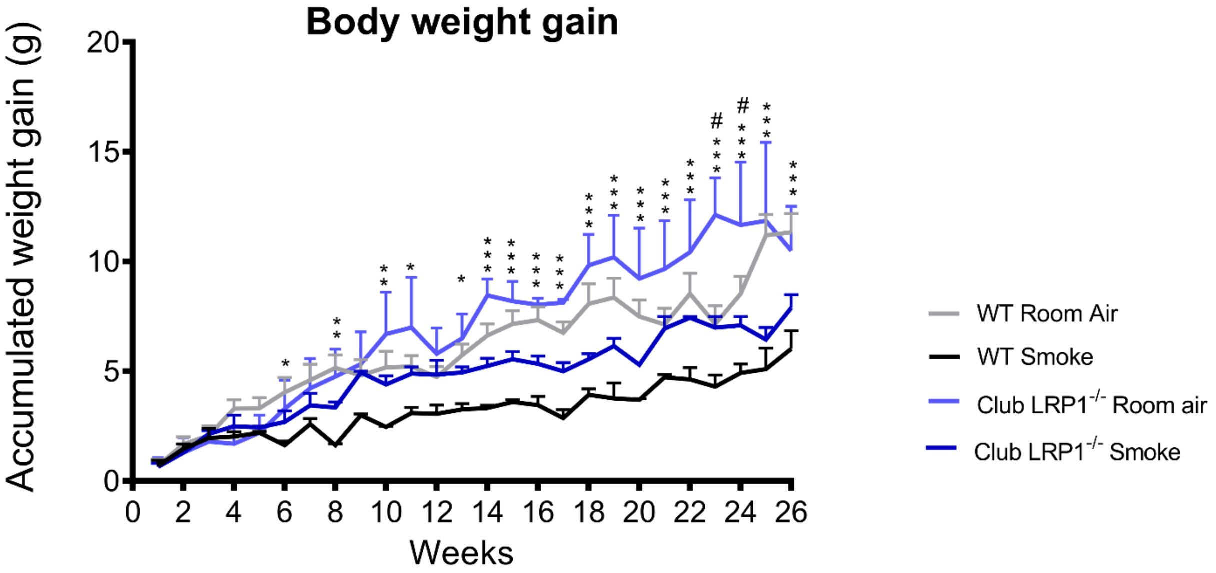

Supplemental Figure S3. Body weight increase in male mice during smoke exposures. Mouse body weight was recorded weekly during the exposure to room-air or smoke. Accumulated weight gain is shown. Statistics: t test: *p<0.05, *p<0.01, vs. smoke exposed (same genotype); #p<0.05 vs. WT (same exposure).

Supplemental Figure S4.

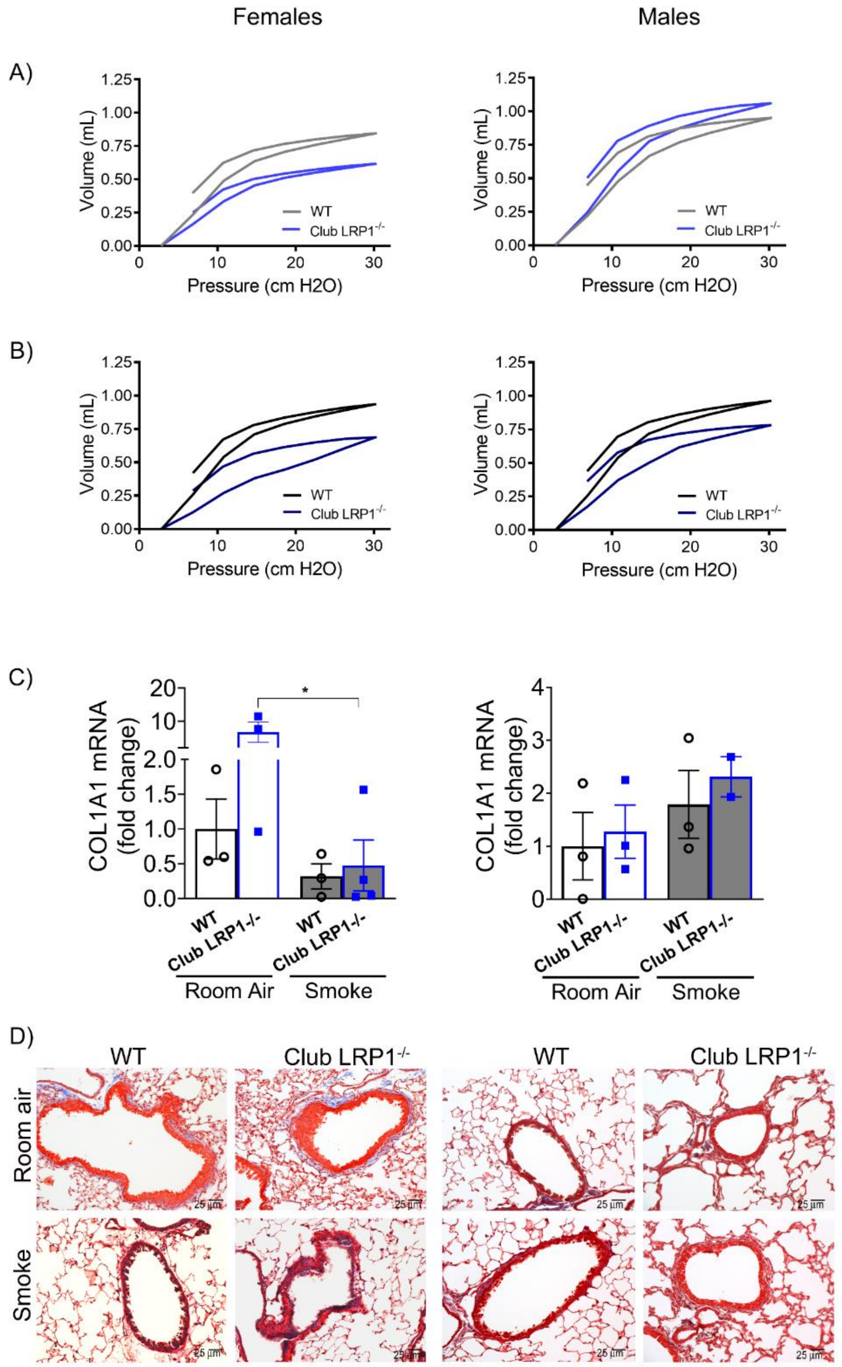

Supplemental Figure S4. Pulmonary function and histology in female and male WT and club *Lrp1*$^{-/-}$ mice. Mice were exposed to smoke or room air for 6 months. Panels in the left side correspond to females, and in the right side to males A) Pressure-volume loops for room air-exposed mice (N= 2-9/condition) B) Pressure-volume loops for smoke-exposed mice, C) Collagen mRNA expression in lung homogenates of WT and club *Lrp1*$^{-/-}$ mice after room air or smoke exposure, D) representative images of Trichrome staining. Panels in the right side correspond to male mice and are shown in Figure 3E too. Panels in the left side correspond to female mice and have been added here to enable side-by-side comparison with male mice. Statistics: 2-way ANOVA followed by Bonferroni post-test. *p<0.05, **p<0.01. ○ WT ■ club *Lrp1*$^{-/-}$

Supplemental Figure S5.

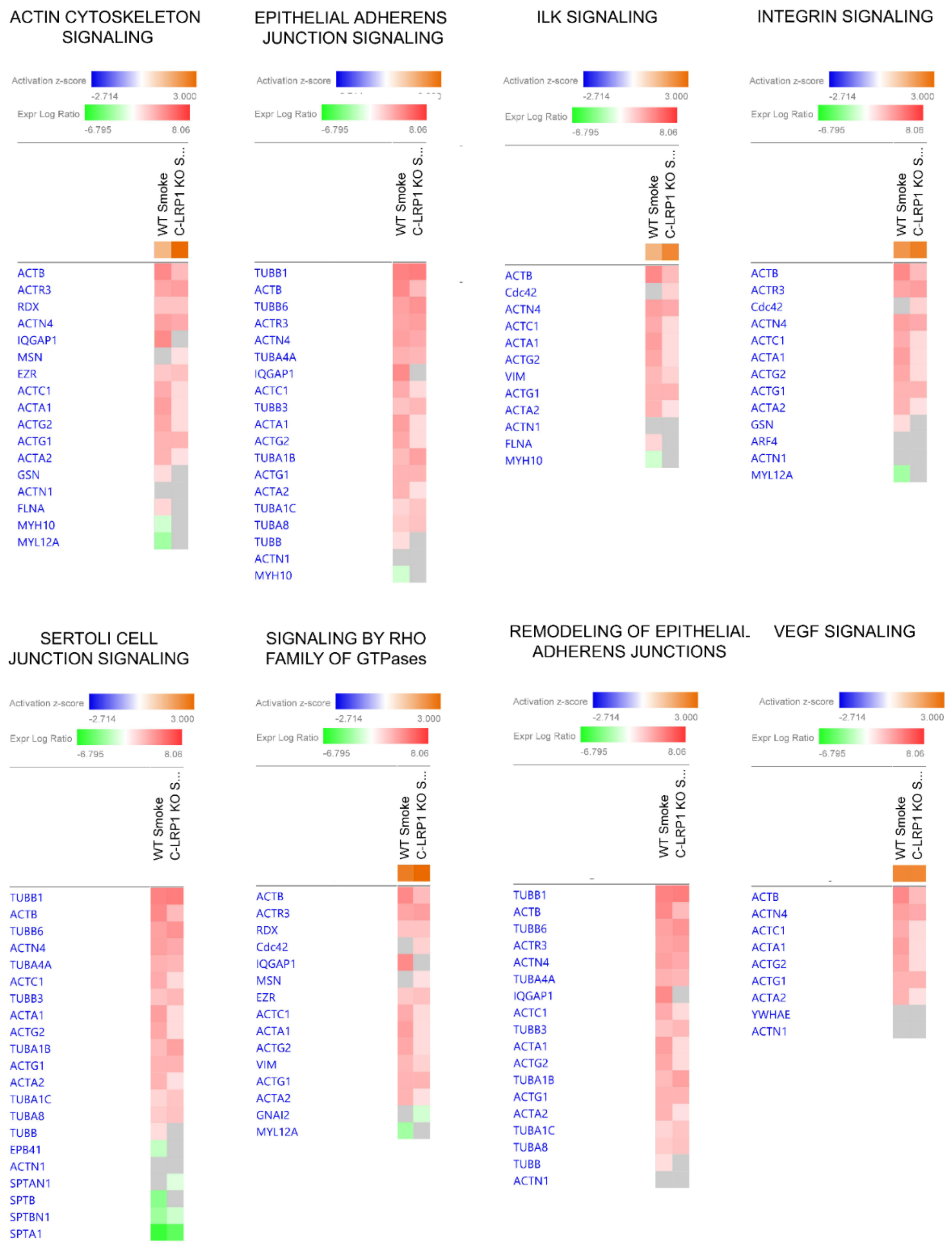

Supplemental Figure S5. Cytoskeleton-related pathways in smoke-exposed WT and club *Lrp1*$^{-/-}$ mice. Protein expression of individual proteins in green-red gradient relative to WT mice exposed to room air. Proteins are listed in order of higher to lower expression and each protein may be included in more than one pathway. Predicted pathway activity based on the expression level of the detected proteins is shown as z-score in blue-orange gradient.

Supplemental Figure S6.

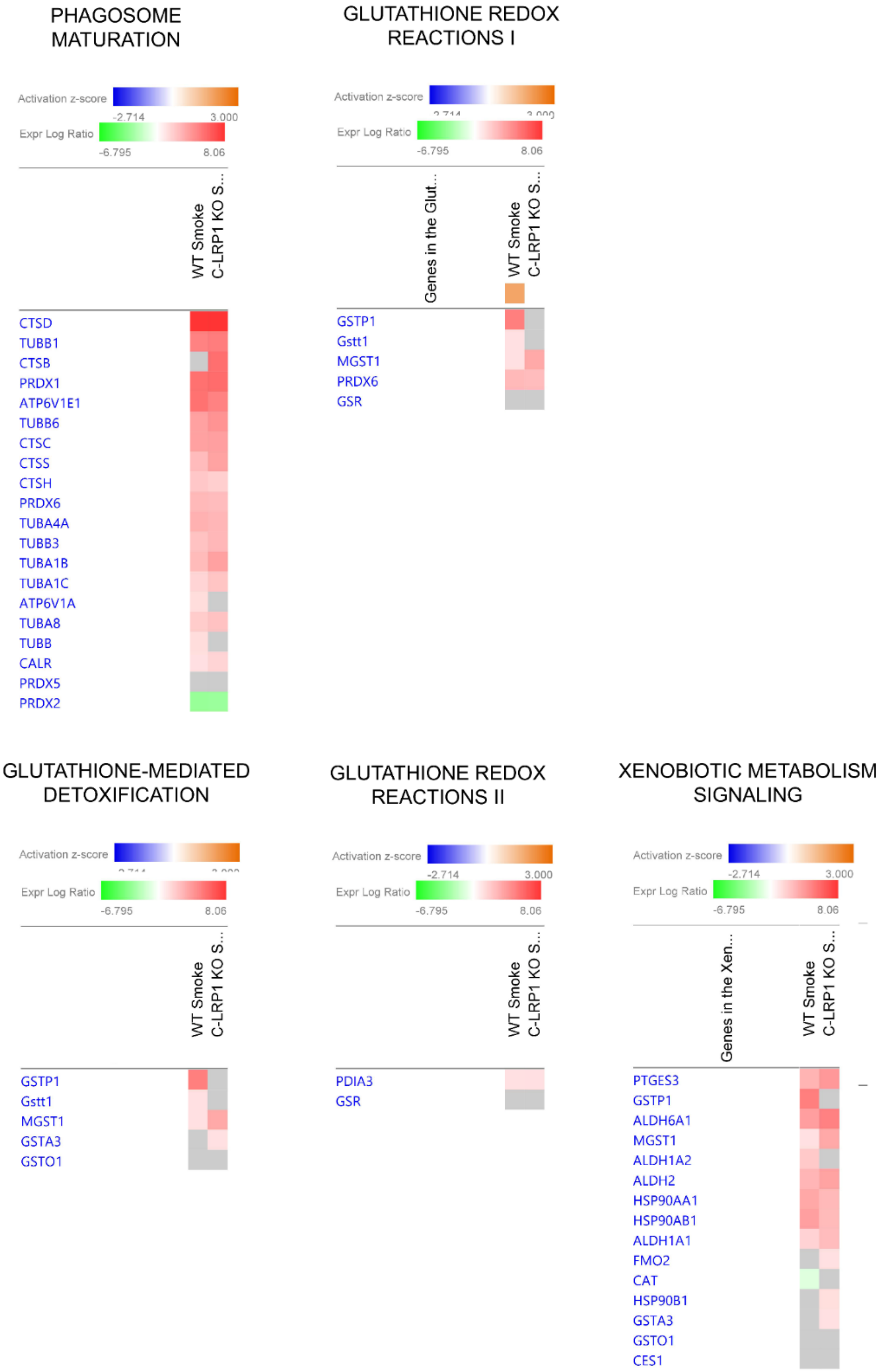

Supplemental Figure S6. Xenobiotic-related pathways in smoke-exposed WT and club *Lrp1*$^{-/-}$ mice. Protein expression of individual proteins in green-red gradient relative to WT mice exposed to room air. Proteins are listed in order of higher to lower expression and each protein may be included in more than one pathway.

Supplemental Figure S7.

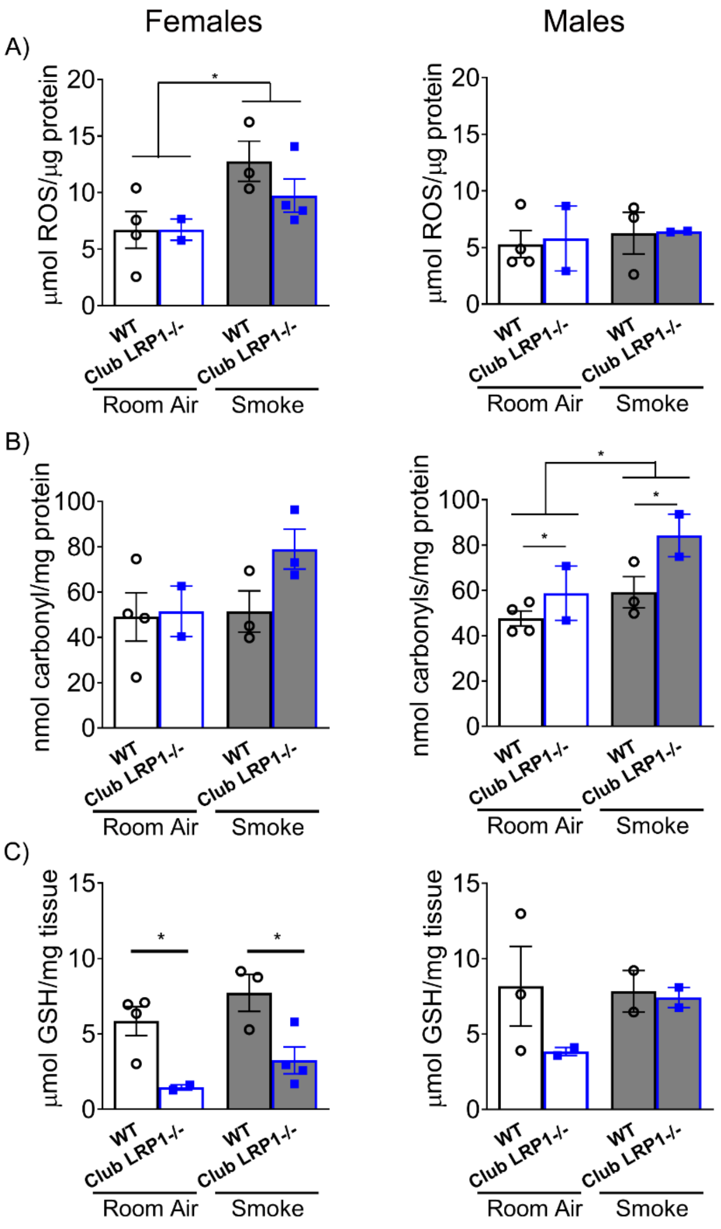

Supplemental figure S7. Analysis of xenobiotic metabolism in WT and club *Lrp1*$^{-/-}$ mice. Panels in the left side correspond to female mice, and in the right to male mice A) Reactive oxygen species (ROS) in whole lungs from WT and club LRP1$^{-/-}$ mice, before and after smoke exposure B) permanent protein oxidation in whole lungs from WT and club LRP1$^{-/-}$ mice before and after smoke exposure. C) total glutathione in whole lungs from WT and club LRP1$^{-/-}$ mice before and after smoke exposure. Statistics: 2-Way ANOVA followed by Bonferroni post-test for panels. *p<0.05. ○ WT ■ club *Lrp1*$^{-/-}$
